# T Cells Promote Metastasis by Regulating Extracellular Matrix Remodeling following Chemotherapy

**DOI:** 10.1158/0008-5472.CAN-21-1012

**Published:** 2021-10-19

**Authors:** Jozafina Haj-Shomaly, Avital Vorontsova, Tamar Barenholz-Cohen, Oshrat Levi-Galibov, Mahesh Devarasetty, Michael Timaner, Ziv Raviv, Tim J. Cooper, Shay Soker, Peleg Hasson, Daphne Weihs, Ruth Scherz-Shouval, Yuval Shaked

**Affiliations:** 1 Rappaport Technion Integrated Cancer Center, Cell Biology and Cancer Science, Rappaport Faculty of Medicine, Technion–Israel Institute of Technology, Haifa, Israel.; 2 Faculty of Biomedical Engineering, Technion–Israel Institute of Technology, Haifa, Israel.; 3 Department of Biomolecular Sciences, Weizmann Institute of Science, Rehovot, Israel.; 4 Wake Forest University School of Medicine, Wake Forest Institute for Regenerative Medicine, Winston-Salem, North Carolina.; 5 Department of Genetics and Developmental Biology, Rappaport Faculty of Medicine, Technion-Israel Institute of Technology, Haifa, Israel.

## Abstract

Metastasis is the main cause of cancer-related mortality. Despite intense efforts to understand the mechanisms underlying the metastatic process, treatment of metastatic cancer is still challenging. Here we describe a chemotherapy-induced, host-mediated mechanism that promotes remodeling of the extracellular matrix (ECM), ultimately facilitating cancer cell seeding and metastasis. Paclitaxel (PTX) chemotherapy enhanced rapid ECM remodeling and mechanostructural changes in the lungs of tumor-free mice, and the protein expression and activity of the ECM remodeling enzyme lysyl oxidase (LOX) increased in response to PTX. A chimeric mouse model harboring genetic LOX depletion revealed chemotherapy-induced ECM remodeling was mediated by CD8^+^ T cells expressing LOX. Consistently, adoptive transfer of CD8^+^ T cells, but not CD4^+^ T cells or B cells, from PTX-treated mice to naïve immunodeprived mice induced pulmonary ECM remodeling. Lastly, in a clinically relevant metastatic breast carcinoma model, LOX inhibition counteracted the metastasis-promoting, ECM-related effects of PTX. This study highlights the role of immune cells in regulating ECM and metastasis following chemotherapy, suggesting that inhibiting chemotherapy-induced ECM remodeling represents a potential therapeutic strategy for metastatic cancer.

**Significance:**

Chemotherapy induces prometastatic pulmonary ECM remodeling by upregulating LOX in T cells, which can be targeted with LOX inhibitors to suppress metastasis.

*See related commentary by Kolonin and Woodward, p. 197*

## Graphical Abstract

**Figure:**
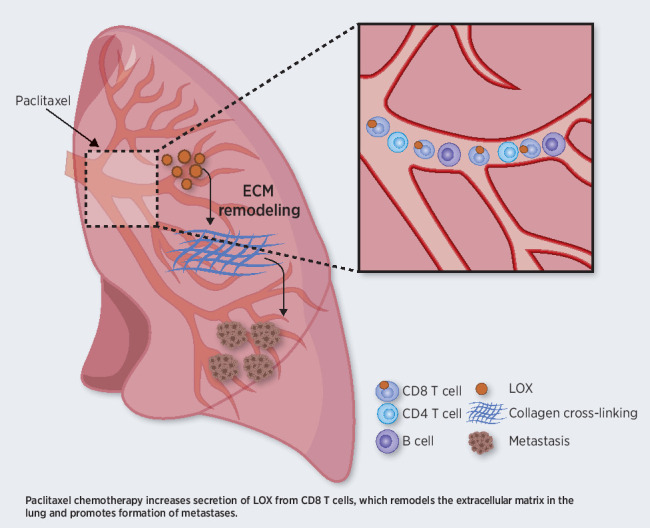

## Introduction

Metastasis is the main cause of cancer-related deaths worldwide including in breast cancer (1). It is a multistep process involving the spread of cancer cells from their primary site to distant organs (2). The process requires cancer cell escape from the primary tumor, invasion into the surrounding stroma, intravasation into blood or lymphatic vessels, and survival in the circulation. In the final steps, cancer cells exit the vessel (extravasation) and seed in a distant organ where they proliferate and form a secondary tumor (1, 2).

Cancer cells do not randomly invade secondary metastatic sites. Rather, they preferentially seed in specific organs that selectively support metastatic colonization (3). The formation of a premetastatic niche is regulated by the dynamic interplay between cancer cells, stromal cells, and the extracellular matrix (ECM; refs. 4, 5). For example, hematopoietic progenitor and myeloid cells home to the premetastatic site where they cluster and create a structural niche for cancer cell seeding (3, 6). In the context of metastasis, an association has been demonstrated between stromal cells and ECM components, such as fibronectin, collagen, versican, periostin, and tenascin C (7, 8). Specifically, increased fibronectin expression in premetastatic tissue is associated with bone marrow–derived cell (BMDC) recruitment (9). These events are thought to prime secondary organ sites before the arrival of cancer cells, weeks before metastases form (3, 10).

The ECM is composed of core structural macromolecules such as collagens, elastin, fibronectin, and laminin. It stores growth factors and bioactive molecules such as matrix metalloproteinases (MMP), heparan sulfate, fibroblast growth factor, and urokinase plasminogen activator (uPA; refs. 8, 11). Cancer and stromal cells in the tumor microenvironment (TME) secrete interstitial matrix that support cell proliferation and tumor growth. Specifically, the ECM at the tumor site provides the structural foundation for the tumor tissue, allowing growth, survival, motility, and differentiation (8, 12). It has been shown that ECM proteins act as an anchor and promote cellular adhesion, whereas the fibers of the ECM may serve as migration tracks for cancer cells (13). Furthermore, the ECM can block infiltration of immune cells into the tumor, as well as prevent the perfusion of anticancer drugs by creating a high interstitial fluid pressure (12). ECM contributes not only to tumor development but also to metastatic spread, serving as the soil for cancer cells to seed. Increased ECM depositions and remodeling require the activity of specific factors and enzymes. Among these enzymes are lysyl oxidase (LOX), cathepsins, and MMPs. LOX is an extracellular copper-dependent amine oxidase that catalyzes collagen and elastin cross-linking, which is essential for stabilization of collagen fibrils and for the integrity and elasticity of elastin (14). LOX secretion by hypoxic cancer cells contributes to the formation of a premetastatic niche and promotes cancer cell seeding in distant organs by enhancing the recruitment of myeloid cells (15). Although these effects have been described in the context of growing tumors, the effect of anticancer drugs on ECM-associated enzymes and their effects on metastasis are still elusive.

We and others have recently demonstrated that anticancer treatments including chemotherapy, radiation, surgery and targeted drugs induce host-mediated protumorigenic and prometastatic effects explaining, in part, tumor relapse and metastasis (for review refs. 16–18). For example, in an experimental lung metastasis model, mice treated with paclitaxel (PTX) chemotherapy and subsequently injected with cancer cells exhibited an increased mortality rate when compared with control mice (19). We found that PTX chemotherapy induces the homing of BMDCs to tumors where they secrete MMP9. In turn, MMP9 induces epithelial-to-mesenchymal transition of cancer cells at the primary tumor site, leading to their dissemination and subsequent seeding in the lungs (19). A recent clinical study reported that chemotherapy enhances the risk for metastasis in the neoadjuvant setting in breast cancer patients. Specifically, tumor microenvironment of metastasis (TMEM), a structure consisting of myeloid, endothelial, and cancer cells, generates new doorways for cancer cells to escape the primary tumor (20). Thus, these studies demonstrate that there are circumstances in which chemotherapy may lead to increased metastasis despite its therapeutic activity. However, little is known about the effect of chemotherapy on the secondary metastatic sites.

Here we show that PTX chemotherapy modulates the ECM in the lungs shortly after treatment, thereby promoting cancer cell seeding and metastasis. We demonstrate that such effects are dependent on LOX-secreting CD8^+^ T cells, and that LOX inhibition counteracts the prometastatic activity of PTX chemotherapy. This study highlights the interplay between immune cells and the ECM in the context of chemotherapy-induced metastasis in breast carcinoma.

## Materials and Methods

### Cell lines

EMT6 murine breast carcinoma, 4T1 murine mammary adenocarcinoma, and MCF7 human breast carcinoma cell lines were purchased from the American Type Culture Collection, and were used within 6 months after resuscitation. The EMT6-F2 cell line, a metastatic variant of EMT6, was generated in our lab in a similar way that was previously described for MDA-MB-231 human breast carcinoma cells (21). All cell lines were cultured in Dulbecco's modified Eagle medium (Sigma-Aldrich) supplemented with 10% fetal bovine serum (Biological Industries), 1% L-glutamine, 1% sodium pyruvate, and 1% streptomycin–penicillin–neomycin solution (Biological Industries). Cells were cultured at 37°C in a humidified atmosphere containing 5% CO_2_. Some of the cell lines were stably transfected with a GFP-expressing vector (Clontech Laboratories, 632379). Cells were routinely tested to be *Mycoplasma*-free using EZ-PCR mycoplasma test kit (Biological industries).

### Drugs and drug concentrations

BALB/c mice, 8 to 10 weeks of age, were injected with 25 mg/kg PTX (Teva), considered to be the maximum tolerated dose (MTD; ref. 22). In some experiments, PTX was administered every 3 weeks, for 3 consecutive cycles, or at a lower dose (10 mg/kg). After 24 or 72 hours, as indicated in the text, blood was drawn by cardiac puncture using heparin tubes, and plasma was separated. Plasma was intraperitoneally injected into recipient mice at a volume of 100 μL/mouse. In other experiments, rabbit anti-LOX antibodies were generated by GenScript, as previously described (23). LOX activity was inhibited with β-aminopropionitrile (BAPN; Sigma-Aldrich) administered intraperitoneally at a daily dose of 100 mg/kg or with LOX neutralizing antibodies administered peritoneally at a dose of 25 μg/kg, as previously described (23). LOX-depleted plasma used in some *in vitro* experiments was prepared as follows. Anti-LOX antibodies (1 μg) were added to 500 μL plasma drawn from BALB/c control- or PTX-treated mice. The mix was incubated for 1 hour with rotation at 4°C. Antibodies were then depleted from the plasma using a mix of protein A/G sepharose beads (Abcam, ab193262).

### Animal models

The use of animals and experimental protocols were approved by the Animal Care and Use Committee of the Technion. EMT6, 4T1, or EMT6/F2 (0.5 × 10^6^) cells were implanted in the mammary fat pad of 8- to 10-week-old BALB/c female mice (Envigo). Tumor size was assessed regularly with Vernier calipers using the formula: width^2^ × length × 0.5.

*LOX^flox/flox^* mice (24) were bred with UBC-CRE-ERT2 (*CRE^+/−^*) mice kindly provided by Prof. Ruby Shalom-Feuerstein (Technion, Israel) to generate ubiquitously inducible LOX-depleted mice upon tamoxifen induction (*CRE^+/−^;LOX^flox/flox^* and their counterpart control *CRE^−/−^;LOX^flox/flox^*). Of note, complete knockout of LOX is lethal. BMDCs of *CRE^+/−^;LOX^flox/flox^* or *CRE^−/−^;LOX^flox/flox^* were harvested from femurs by flushing. BMDCs were then transplanted into lethally irradiated mice (10 Gy total body radiation, at a dose rate of 125 cGy per minute). Two months following the bone marrow transplantation, tamoxifen was administered intraperitoneally at a daily dose of 100 mg/kg for 5 consecutive days. Two days later, mice were treated with PTX or vehicle control and sacrificed 3 days later. Lungs and bone marrow were harvested for further analysis.

In some experiments, *in vivo* lung metastasis assay was performed. Briefly, 8- to 10-week-old BALB/c or SCID mice were treated with PTX or vehicle control. After 72 hours the mice were intravenously injected through the tail vein with EMT6 cells (5 × 10^4^ cells/mouse) tagged with luciferase. The mice were monitored over time using IVIS. After 2 weeks, mice were sacrificed and lungs were removed, sectioned and stained with hematoxylin and eosin (H&E) to detect metastatic foci.

For the adoptive transfer experiments performed with CD8^+^ T cells, CD4^+^ T cells, or B cells, single-cell suspensions were prepared from spleens harvested from control- or PTX-treated BALB/c mice. Cells were immunostained with anti-CD8^+^, anti-CD4^+^, and B220^+^ antibodies (BioLegend) and sorted using Melody sorter. Next, the collected cells (5 × 10^6^ per mouse) were intravenously injected into 8-week-old naïve SCID mice (Envigo). Three days following the adoptive transfer, the mice were sacrificed and lungs were removed for further analysis. All *in vivo* experiments were repeated at least twice.

### Flow cytometry

Lung and spleen samples were prepared as single-cell suspensions as previously described (19, 25). More details are provided in Supplementary Data.

### Immunostaining

Frozen lung tissues were immunostained as previously described (19). More details are provided in Supplementary Data.

### Cell adhesion assay

Cell adhesion to ECM protein-coated substrates was evaluated using a centrifugation assay (26). More details are provided in Supplementary Data.

### *Ex vivo* pulmonary metastatic assay

The assay was performed as previously described (27). More details are provided in Supplementary Data.

### Newly synthesized collagen assay

Newly synthesized collagen in lung lysates was quantified using the Sircol collagen assay kit (Biocolor) in accordance with the manufacturer's instructions. Briefly, Sircol dye reagent was added to lung lysates followed by agitation in a mechanical shaker for 30 minutes. Then, the mix was centrifuged at 12,000 × *g* for 10 minutes. The pellet was washed with acid-salt wash reagent and centrifuged again. Alkali reagent was then added, and the light absorption of the samples was measured at 555 nm wavelength using Infinite 200PRO plate reader (Tecan). Collagen concentration was calculated according to a standard curve. Results were normalized according to the protein concentration in lung extracts. The experiment was performed in biological triplicates.

### Second harmonic generation imaging

Frozen blocks of lungs were sliced at a thickness of 100 μm in PBS and were processed for the analysis of collagen structure as previously described (23). More details are provided in Supplementary Data.

### LOX activity assay

LOX activity was evaluated as previously described (23). More details are provided in Supplementary Data.

### Real-time quantitative PCR

RNA was extracted from the lungs of mice using the total RNA purification kit (Norgen Biotek) in accordance with the manufacturer's protocol. Complementary DNA (cDNA) was then synthesized from the mRNA samples using High-Capacity cDNA Reverse Transcription Kit (Applied Biosystems, CA). Real-time quantitative PCR (RT-qPCR) reaction was performed using SYBR Green Master Mix and run in CFX Connect Real-Time PCR Detection System (Bio-Rad Laboratories). Analysis was performed using the ΔΔC_t_ method. Primers are listed in Supplementary Table S1.

### Rheometry of tissue slices

The assay was performed as previously described (28). More details are provided in Supplementary Data.

### Heparanase activity assay

Preparation of ECM-coated 35 mm dishes and determination of heparanase activity were performed as previously described (29). More details are provided in Supplementary Data.

### Focal adhesion assay

Plasma from control- or PTX-treated mice was incubated for 4 hours on plates coated with fibronectin (20 μg/mL), laminin (20 μg/mL), or collagen type I (20 μg/mL). Then, the plasma was washed, and MCF7 cells (5 × 10^5^/well) were seeded and incubated overnight at 37°C. Next, the cells were collected and lysed to evaluate expression of paxillin and phospho-paxillin using Western blotting.

### Statistical analysis

For adequate statistical power, all experiments were performed with at least three biological repeats and two technical repeats. In the *in vitro* and immunostaining studies, analysis was performed on at least three biological repeats and >4 fields/group were assessed. The *in vivo* experiments were repeated at least twice, with number of mice indicated in the figure legends (usually *n* = 3–5 mice/group). All experiments were performed in a randomized manner. Data are presented as mean ± SE. Statistically significant differences were assessed by one-way ANOVA, followed by Tukey *post hoc* test (when comparing between more than two groups) using GraphPad Prism 5 software. When applicable, estimate of variance was performed and statistical significance comparing only two sets of data was determined by two-tailed Student *t* test. Significance was set at *P* < 0.05, and designated as follows: *, *P* < 0.05; **, *P* < 0.01; ***, *P* < 0.001.

## Results

### Chemotherapy enhances pulmonary ECM remodeling

**Figure 1.**
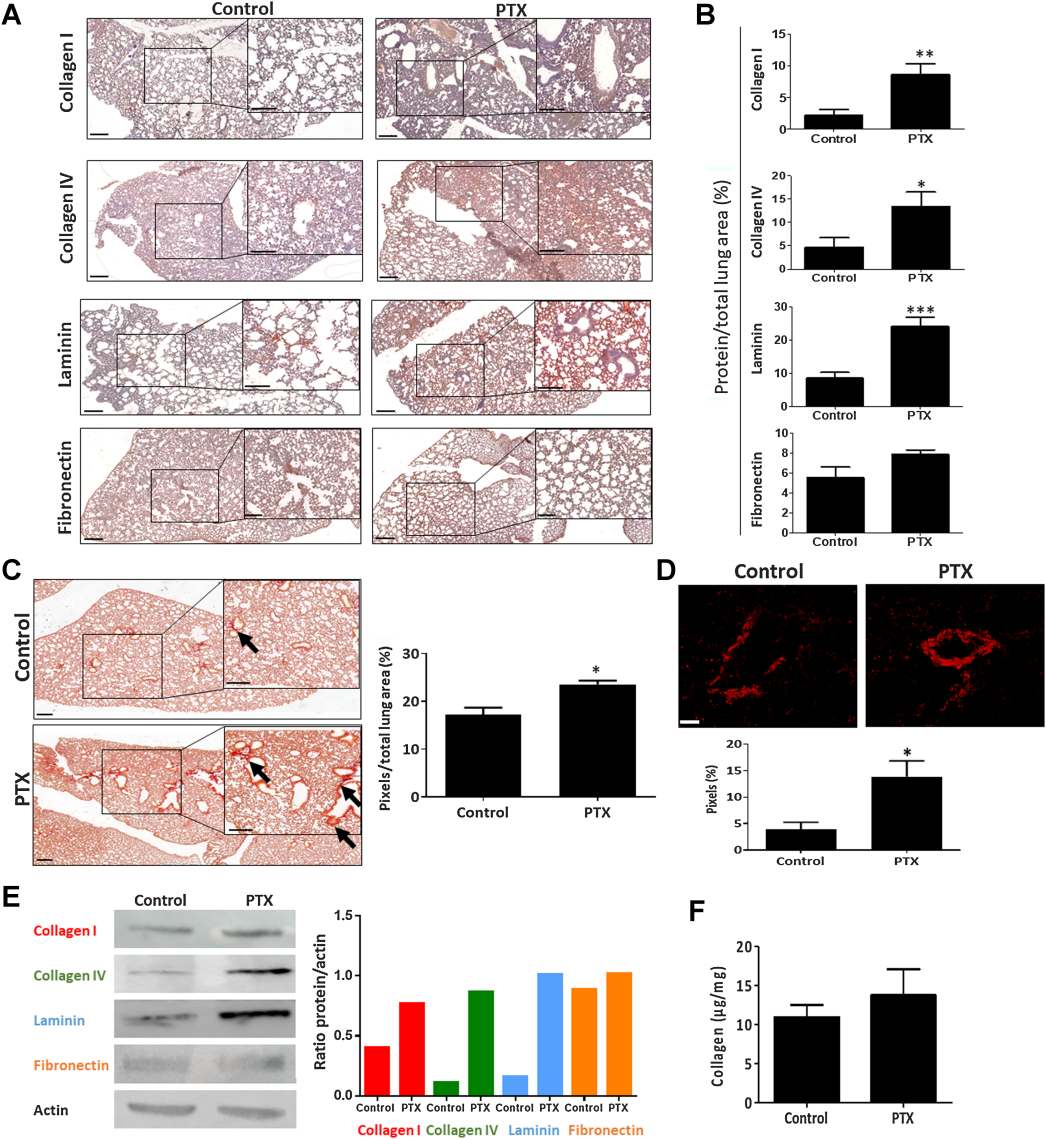
Chemotherapy induces pulmonary ECM remodeling. Tumor-free, 8- to 10-week-old BALB/c mice were treated with PTX or vehicle (control). After 72 hours, mice were sacrificed and lungs were removed. Lungs were formalin-fixed and subsequently sectioned (*n* = 5 mice/group). **A,** Lung sections were immunostained with antibodies against collagen I, collagen IV, laminin, and fibronectin (shown in brown) and counterstained with hematoxylin. Representative images in two magnifications are shown. Scale bar, 200 μm. **B,** The percentage of positive pixels per total lung area was quantified. **C,** Lung sections were stained with Sirius red to detect collagen and elastin (red). Representative images in two magnifications are shown. Arrows indicate collagen and elastin staining. Collagen and elastin levels were quantified by calculating the percentage of red pixels per total lung area. Scale bar, 200 μm. **D,** Lung cryo-sections from the control and 72 hours after PTX therapy (PTX) were imaged by 2-photon microscopy. Top, representative SHG images are shown. Fibrillar collagen structure is designated in red. Scale bar, 50 μm. Bottom, the percentage of red pixels was quantified (*n* > 4 fields/lung). **E** and **F,** In a parallel experiment, protein lysates from lungs obtained from mice 72 hours after treatment with vehicle control or PTX (*n* = 5 mice/group) were analyzed for the expression of the indicated ECM components by Western blot, followed by densitometry analysis (**E**) or for newly synthesized collagen, using Sircol collagen assay kit (**F**). Statistical significance was assessed by unpaired two-tailed *t* test. Significant *P* values are shown as *, *P* < 0.05; **, *P* < 0.01; ***, *P* < 0.001 from control.

An induction of fibrosis at secondary metastatic sites in mouse models of cancer has been previously described as a process that favors metastasis (30). However, it is currently not known whether chemotherapy elicits acute metastasis-supporting changes in the ECM and whether these effects are tumor- or host-dependent. To investigate this, we treated tumor-free mice with PTX chemotherapy and analyzed the changes in pulmonary ECM components over time. Of note, the use of tumor-free mice allows the detection of host-mediated effects alone. Substantial and significant increases in the levels of collagen I, collagen IV, and laminin were observed in response to PTX, reaching the highest peak at the 72-hour time point for all tested proteins as observed by immunofluorescence and immunohistochemistry staining (Fig. 1A and B; Supplementary Fig. S1A and S1B). These effects were also observed when using Masson's trichrome staining (Supplementary Fig. S1C and S1D). In addition, Sirius red staining, second harmonic microscopy images, and Western blot analyses of different ECM proteins from lungs of PTX-treated mice confirmed ECM structural changes 72 hours after PTX treatment (Fig. 1C–E). Of note, when comparing control and PTX groups, no noticeable differences in collagen expression were detected in liver and spleen tissue at the 72-hour time point, indicating that PTX elicits these effects specifically in the lungs (Supplementary Fig. S1E and S1F). Importantly, similar effects were observed in lungs of PTX-treated mice bearing EMT6 or 4T1 breast carcinomas, suggesting that tumor presence does not significantly affect ECM remodeling in this experimental setup (Supplementary Fig. S2).

We next evaluated whether this ECM remodeling is due to transcriptional changes or posttranslational modifications. RT-qPCR on total RNA extracted from lungs of control- or PTX-treated mice revealed no significant changes in mRNA levels of collagen I, collagen IV, laminin, and fibronectin (Supplementary Fig. S3). Similarly, there was no significant change in the level of newly synthesized collagen assessed by Sircol assay in the lungs of PTX-treated mice (Fig. 1F), suggesting that ECM remodeling is associated with posttranslational modification. Furthermore, it should be noted that mice treated with several cycles of PTX chemotherapy or with a lower dose of PTX displayed comparable pulmonary ECM changes to those observed in mice treated with single, high-dose PTX (Supplementary Fig. S4). Taken together, these results indicate that the rapid changes in ECM architecture in response to chemotherapy are associated with ECM remodeling rather than synthesis, an effect that is primarily mediated by the host.

### Chemotherapy-induced changes in pulmonary ECM facilitate cancer cell seeding

**Figure 2.**
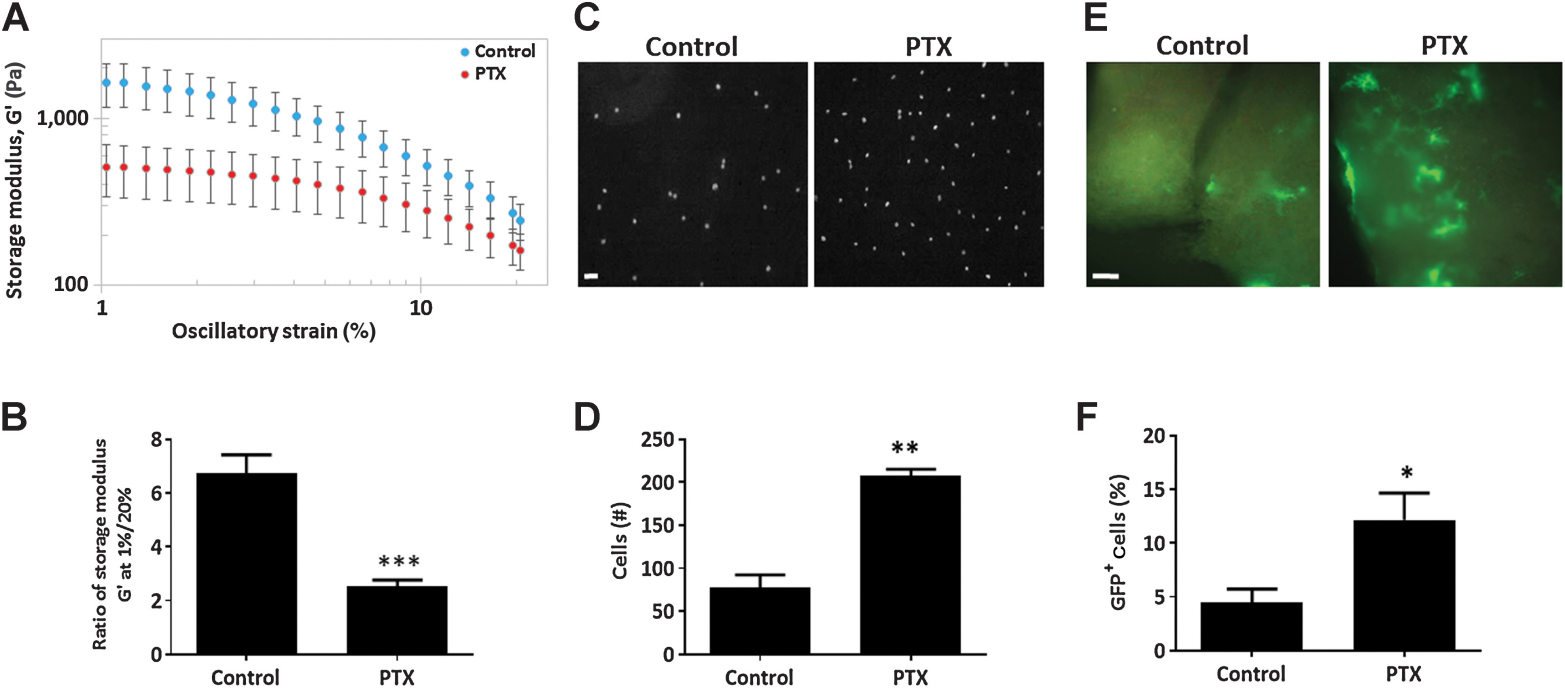
Chemotherapy-induced ECM remodeling promotes cancer cell seeding. **A** and **B,** Tumor-free, 8- to 10-week-old BALB/c mice were treated with PTX or vehicle control (*n* = 5 mice/group). Lungs were removed after 72 hours, and their mechanical response to oscillatory strains was evaluated (**A**). The ratio of the storage (elastic) modulus between low and high strain levels is shown (**B**). **C** and **D,** In a parallel experiment, lysates were prepared from the lungs of control- and PTX-treated mice (*n* = 5 mice/group). Collagen-coated plates were pretreated with lung lysates (100 μg) for 4 hours, followed by washing. EMT6 cells (2 × 10^4^ cells/well) were then seeded onto the plates, and after 15 minutes, nonadherent cells were removed. Adherent cells were stained with DAPI and analyzed by fluorescence microscopy. Representative images are shown in **C**. Scale bar, 60 μm. Quantification of adherent cells is shown in **D**. **E** and **F,** Tumor-free, 8- to 10-week-old BALB/c mice were treated with PTX or vehicle control (*n* = 4 mice/group). After 72 hours, EMT6-GFP^+^ cancer cells (25 × 10^4^ cells/mouse) were injected through the tail vein to generate an *ex vivo* PuMA. After 15 minutes, lungs were perfused, excised, and sectioned. Lung sections were cultured for 1 week and analyzed by FluorVivo Mag system. Representative images are shown in **E**. Scale bar, 200 μm. The percentage of GFP^+^ cells in single-cell suspensions of lung sections was analyzed by flow cytometry (**F**). Statistical significance was assessed by an unpaired two-tailed *t* test. Significant *P* values are shown as *, *P* < 0.05; **, *P* < 0.01; ***, *P* < 0.001.

We next sought to determine whether chemotherapy-induced ECM changes affect cancer cell seeding in the lungs, thereby supporting metastasis. We first studied the mechanical characteristics of the ECM. To this end, lungs were removed from control- and PTX-treated tumor-free mice, and subsequently exposed to different oscillatory strain amplitudes to evaluate the material moduli; i.e., lung-tissue resistance to strain was measured in terms of elastic and viscous response. The lung-tissue deformation was more solid-like or elastic in response, with an average phase angle of ∼25 degrees for both control- and PTX-treated mice; sample response remained more elastic-like under all evaluated strains. Control lung tissues were consistently stiffer than lung tissue from PTX-treated mice, yet the highest tested strains (20%) reduced the stiffness to the same values (Fig. 2A). The reduction in the sample stiffness under applied strain was thus significantly (*P* < 0.002) smaller in the lung-tissue from PTX-treated mice as compared with the controls (Fig. 2B). The deformation of the lung-tissue samples from both the control- and PTX-treated mice was more solid-like or elastic in response to all evaluated strains (1%–20%), which likely results from the response of the ECM network structure in the tissue; the average phase angle of the samples was ∼25 degrees under 1% strains. Thus, although the samples demonstrate likely structural differences in their ECM, the underlying lung structure is largely unchanged.

Next, using an *in vitro* cell adhesion assay, we assessed whether chemotherapy-induced changes in lung tissue affect cancer cell seeding. To this end, collagen-coated plates were primed with lung lysates from control- or PTX-treated mice, followed by the addition of EMT6 cells to the plates. EMT6 cells adhered better to the plates primed with lysates extracted from the lungs of PTX-treated mice in comparison with the control group (Fig. 2C and D). Comparable results were observed when using 4T1 and MCF7 cells (Supplementary Fig. S5). Finally, to determine whether chemotherapy-induced changes in pulmonary ECM promote cancer cell seeding, we performed an *ex vivo* pulmonary metastatic assay (PuMA), as detailed in Supplementary Materials and Methods. A significant increase in the percentage of cancer cells was detected in lung sections from PTX-treated mice in comparison with control. Of note, there was no difference in the size of the growing tumor colonies in the lungs, suggesting that tumor cell proliferation is not affected (Fig. 2E and F). In another experiment, the percentage of tumor cells (EMT6-GFP^+^; 2 × 10^5^ cells) seeded in the lungs 15 minutes after tail-vein injection was evaluated by flow cytometry. A significantly higher percentage of GFP^+^ cells were detected in mice treated with PTX compared with control mice (Supplementary Fig. S6). Taken together, these results suggest that PTX treatment enhances cancer cell seeding in the lungs. Similar effects of increased metastasis in response to PTX therapy were observed in an *in vivo* experimental lung metastasis assay performed on BALB/c mice (Supplementary Fig. S7). Taken together, our findings suggest that PTX treatment induces rapid changes in pulmonary ECM, thereby facilitating cancer cell seeding and promoting metastasis.

### Chemotherapy increases LOX levels and activity in the lungs

**Figure 3.**
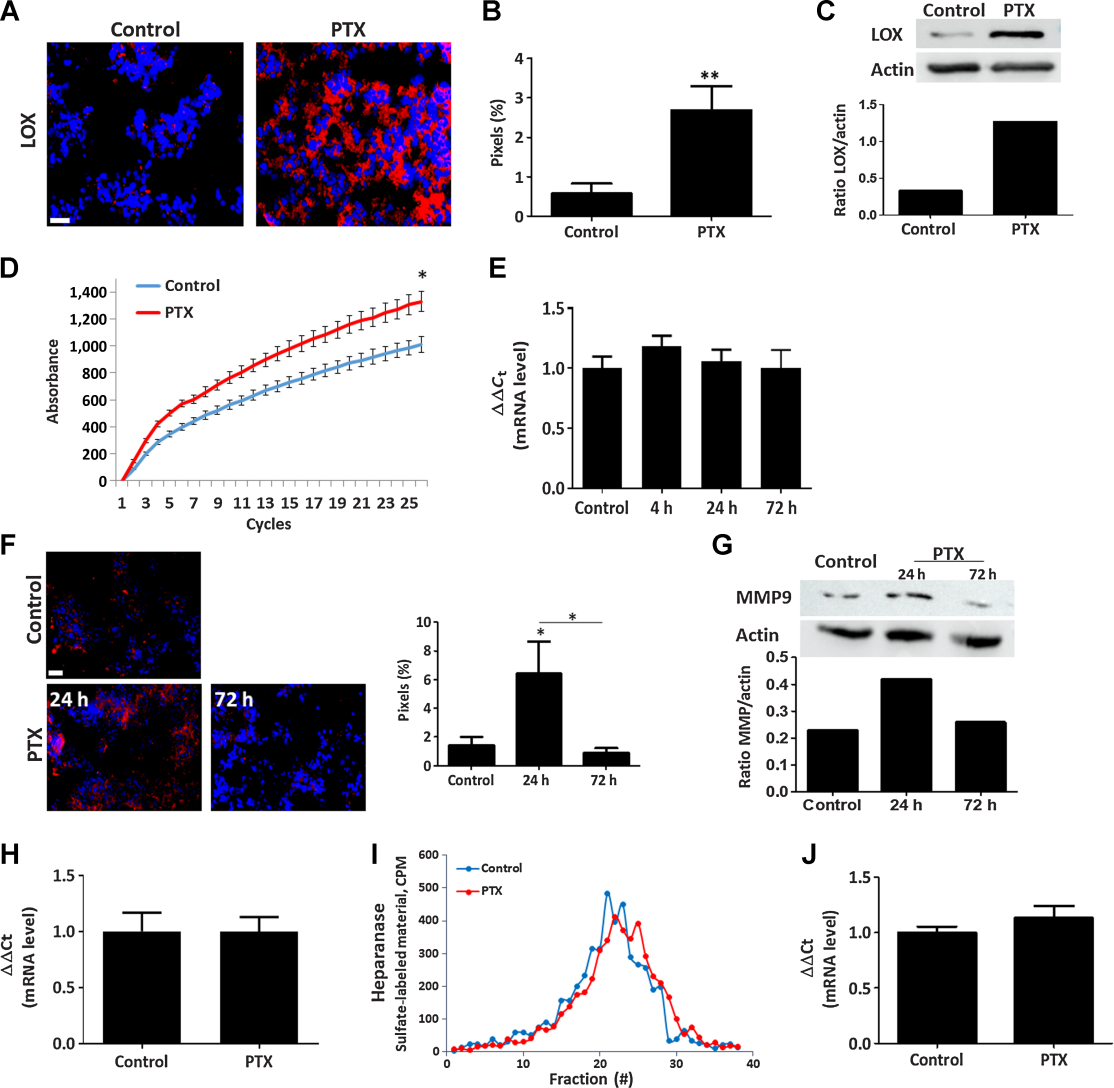
PTX chemotherapy increases LOX levels and activity in the lungs. Tumor-free, 8- to 10-week-old BALB/c mice (*n* = 4 mice/group) were treated with PTX or vehicle control, and lungs were removed after 72 hours. **A,** Lung sections were immunostained with antibodies against LOX (red), and nuclei were stained with DAPI (blue). Representative images are shown. Scale bar, 50 μm. **B,** The percentage of red pixels per field was quantified (*n* > 4 fields/lung). **C,** LOX protein expression in lung extracts was evaluated by Western blot, followed by densitometry analysis. **D,** LOX activity was analyzed in lung lysates. **E,** In a parallel experiment, lungs (*n* = 4 mice/group) were obtained 4 to 72 hours after the mice were treated with PTX or vehicle control. LOX mRNA levels in lung tissue were quantified by RT-qPCR. **F,** Left, lung sections from mice 24 and 72 hours after PTX therapy or from vehicle-treated (control) mice were immunostained with antibodies against MMP9 (red). Nuclei were stained with DAPI (blue). Scale bar, 50 μm. Right, the percentage of red pixels per field was quantified (*n* > 4 fields/lung). **G,** In parallel, lung lysates were analyzed for the expression levels of MMP9 using Western blot, followed by densitometry analysis. **H,** In a separate experiment, mRNA was extracted from the lungs of control- and PTX-treated mice 72 hours after treatment. MMP9 mRNA levels were quantified by RT-qPCR (*n* = 4 biological repeats). **I** and **J,** Lungs were extracted from control- and PTX-treated mice 72 hours after treatment. Heparanase activity in lung lysates was assessed (**I**), and heparanase mRNA levels in lung tissue were quantified by RT-qPCR (**J**). Statistical significance was assessed by one-way ANOVA followed by Tukey posttest when more than two groups were analyzed or unpaired two-tailed *t* test when only two groups were analyzed. Significant *P* values are shown as *, *P* < 0.05; **, *P* < 0.01; ***, *P* < 0.001 from control or otherwise indicated in the figure.

ECM remodeling, i.e., degradation and reassembly of ECM components, occurs via the activity of several catalytic enzymes that act extracellularly (31). Among these enzymes is LOX that plays a critical role in ECM remodeling by catalyzing collagen cross-linking. To determine whether LOX is involved in chemotherapy-induced ECM changes in lung tissue, we evaluated its expression and activity in the lungs of control- and PTX-treated mice. We found that the protein level of LOX (measured by immunofluorescence and Western blot) as well as LOX enzymatic activity were significantly increased in the lungs of PTX-treated mice in comparison with control mice at the 72-hour time point (Fig. 3A–D). Notably, these effects were not associated with changes in the mRNA levels of LOX evaluated at 4, 24, and 72 hours after chemotherapy administration (Fig. 3E). Comparable results of LOX activity, protein expression, and mRNA levels were also observed in the lungs of mice bearing EMT6 or 4T1 tumors (Supplementary Fig. S8).

Next, we analyzed the expression and activity of two other key ECM-modifying enzymes, namely, MMP9 and heparanase (32, 33). No changes were detected in their expression levels, at the protein and mRNA levels or their activity in lung tissue 72 hours following PTX treatment (Fig. 3F–J). Notably, at the 24-hour time point, MMP9 expression was upregulated based on immunofluorescent staining and Western blot analyses (Fig. 3F and G), in line with a previous study demonstrating that BMDCs highly express MMP9, 24 hours after PTX treatment (19). Taken together, our findings suggest that LOX is one of the main enzymes involved in ECM remodeling 72 hours following PTX treatment.

### Chemotherapy-induced ECM remodeling in the lungs occurs via a systemic manner

**Figure 4.**
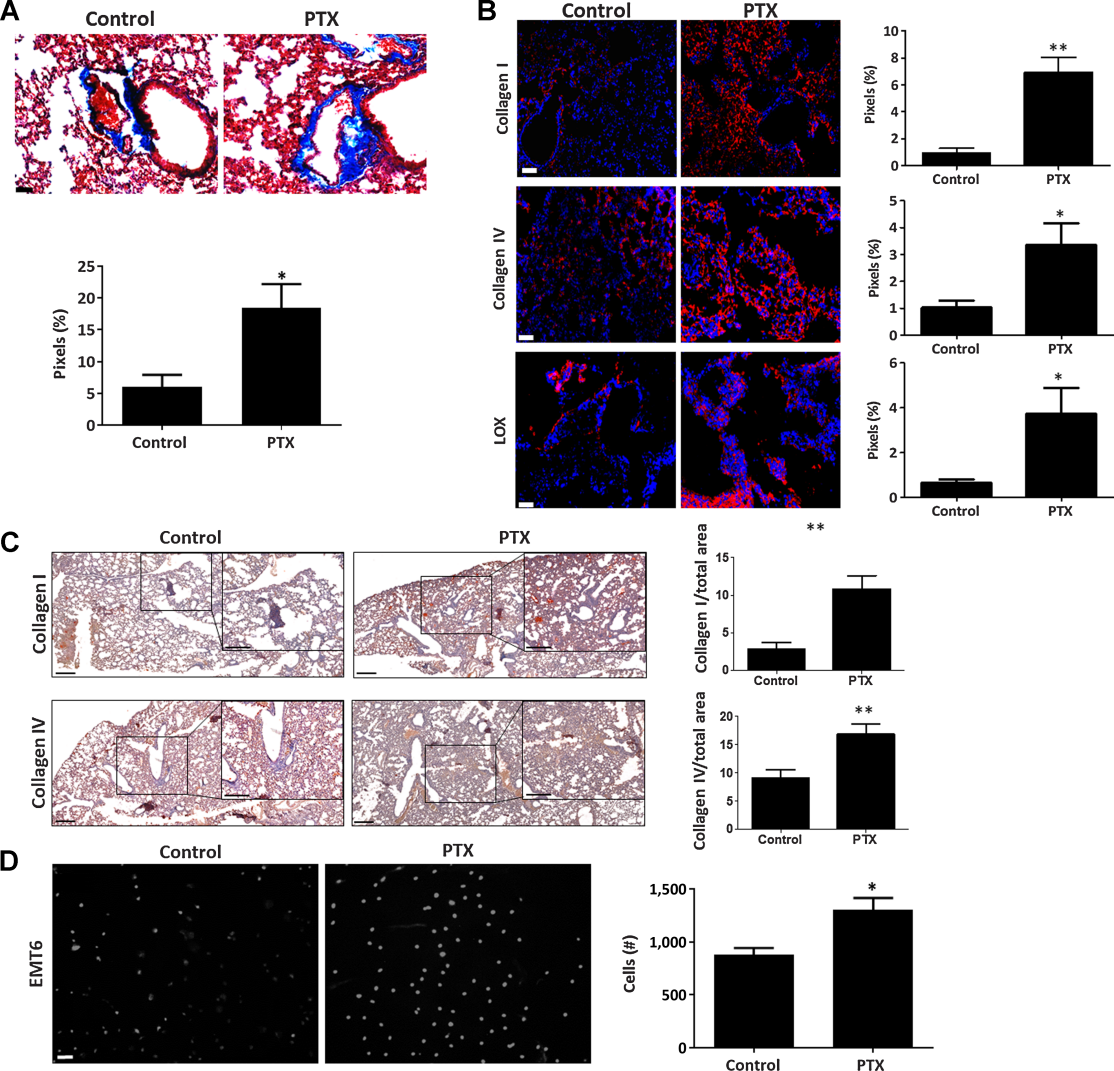
Plasma from PTX-treated mice promotes pulmonary ECM remodeling. Tumor-free, 8- to 10-week-old BALB/c mice were treated with PTX or vehicle control. After 24 hours, blood was obtained by cardiac puncture and plasma was separated. The plasma was then injected intraperitoneally to naïve BALB/c mice (100 μL/mouse), and 72 hours later, lungs were removed (*n* = 4 mice/group). **A,** Lungs were analyzed for collagen content by Masson's trichrome staining. Representative images are shown with quantification of collagen staining per field (*n* > 4 fields/lung). Scale bar, 50 μm. **B,** The levels of collagen I, collagen IV, and LOX in lung sections were analyzed by immunostaining (red). Nuclei were stained with DAPI (blue). Scale bar, 50 μm. The percentage of red pixels per field was quantified (*n* > 4 fields/lung). **C,** Lung sections were also immunostained with antibodies against Collagen I and IV (shown in brown), and counterstained with hematoxylin. Representative images in two magnifications are shown. Scale bar, 200 μm. **D,** In a parallel experiment, collagen-coated plates were pretreated with plasma (10%) from control or PTX-treated mice for 4 hours, followed by washing. EMT6 cells (2 × 10^4^ cells/well) were then seeded onto the plates, and after 15 minutes, nonadherent cells were removed. Adherent cells were stained with DAPI and analyzed by fluorescence microscopy. Representative images are shown on the left. Scale bar, 75 μm. Quantification of adherent cells is shown on the right. Statistical significance was assessed by unpaired two-tailed *t* test. Significant *P* values are shown as *, *P* < 0.05; **, *P* < 0.01; ***, *P* < 0.001.

We next asked whether the increased LOX levels and activity in the lungs following chemotherapy are due to systemic rather than local effects, given that mRNA levels of LOX did not change in the lungs (Fig. 3E). To test this, plasma was obtained from control- or PTX-treated tumor-free mice, 24 hours after drug administration. At this point, negligible traces of PTX are present in the plasma due to its short half-life (19). The plasma was intraperitoneally injected into naïve mice, and lungs were harvested 72 hours later for the analysis of ECM components. Similar to mice directly treated with PTX, the lungs of mice injected with plasma from PTX-treated mice exhibited a significant increase in the levels of collagen I and IV and LOX compared with the lungs from mice injected with plasma from control mice (Fig. 4A–C). These effects were functionally confirmed by the cell adhesion assay, demonstrating that EMT6, 4T1, and MCF7 cells adhered better to collagen I–coated plates primed with plasma from PTX-treated mice (Fig. 4D; Supplementary Fig. S9A and S9B).

To further confirm that cancer cell adhesion is affected by systemic factors induced by PTX treatment, MCF7 cells were seeded on fibronectin, laminin, or collagen I–coated plates that had been previously primed with plasma from control or PTX-treated mice. Of note, in this system, tumor-derived or locally produced LOX are absent because MCF7 cells marginally express LOX (34). An increase in phospho-paxillin expression was found in MCF7 cells seeded on each of the ECM components when the plates were primed with plasma from PTX-treated mice compared with plasma from control mice (Supplementary Fig. S9C). Collectively, these findings suggest that PTX treatment induces systemic effects that ultimately promote rapid pulmonary ECM remodeling and facilitate cancer cell adhesion activity.

### Lymphocytes account for chemotherapy-induced ECM remodeling

**Figure 5.**
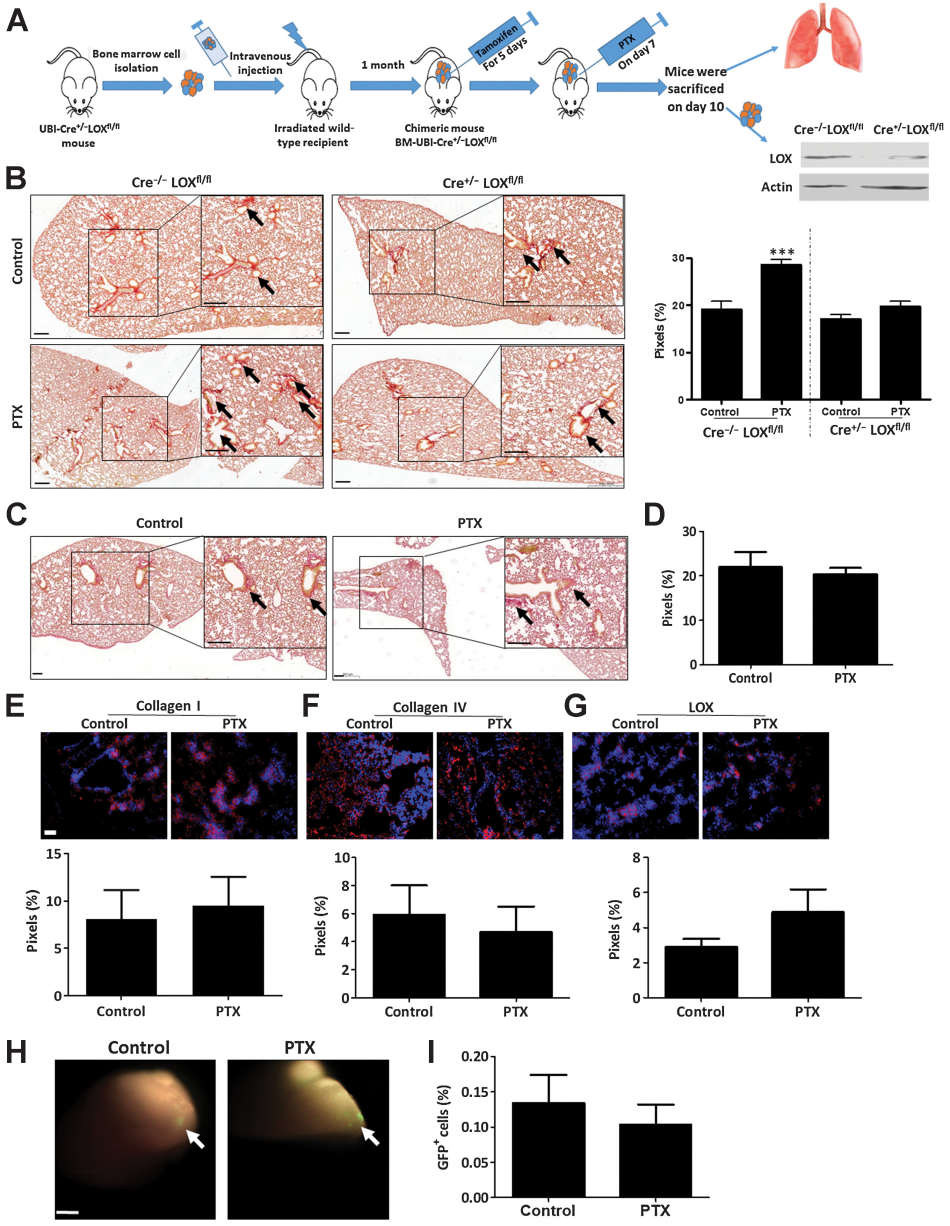
Systemic LOX promotes ECM remodeling following PTX chemotherapy. Bone marrow cells were flushed from Cre^+/−^LOX^flox/flox^ or their control counterpart Cre^−/−^LOX^flox/flox^ mice. The bone marrow cells were then implanted into lethally irradiated wild-type BALB/c mice to generate a chimeric mouse model of inducible LOX depletion specifically in bone marrow cells. The chimeric mice (*n* = 6 mice/group) were treated with tamoxifen for 5 days, and PTX was administered on day 7. After 72 hours, mice were sacrificed and lungs and bone marrow were harvested. **A,** A schematic of the experimental procedure is presented. LOX levels in bone marrow cells were assessed by Western blot. **B,** Lung sections were stained with Sirius red to detect both collagen and elastin. Representative images are shown in two magnifications. Arrows, positive staining. Scale bar, 200 μm. Collagen and elastin levels were quantified by calculating the percentage of red pixels per field (*n* > 4 fields/lung). **C–G,** Tumor-free, 8- to 10-week-old SCID mice (*n* = 4–5 mice/group) were treated with PTX or vehicle control. After 72 hours, lungs were removed. Collagen and elastin in lung sections were detected by Sirius staining. Representative images in two magnifications are shown in **C** (scale bar, 200 μm), and quantification is shown in **D**. Lung sections were immunostained using antibodies against Collagen I (**E**), Collagen IV (**F**), and LOX (**G**). Nuclei were stained with DAPI (blue). Representative images (scale bar, 50 μm) and quantifications (*n* > 4 fields/lung) are shown. **H** and **I,** Tumor-free, 8- to 10-week-old SCID mice (*n* = 5–6 mice/group) were treated with PTX or vehicle control. After 72 hours, EMT6-GFP^+^ cells (25 × 10^4^ cells/mouse) were injected through the tail vein to generate an *ex vivo* PuMA. After 15 minutes, lungs were perfused, excised, and sectioned. Lung sections were cultured for 1 week and analyzed by FluorVivo Mag system. Representative images are shown in **H**. Scale bar, 200 μm. The percentage of GFP^+^ cells in single-cell suspensions of lung sections was analyzed by flow cytometry (**I**). Statistical significance was assessed by one-way ANOVA followed by Tukey posttest when more than two groups were analyzed or unpaired two-tailed *t* test when only two groups were analyzed. Significant *P* values are shown as *, *P* < 0.05; **, *P* < 0.01; ***, *P* < 0.001 from control or otherwise indicated in the figure.

We next sought to determine the cellular source from which chemotherapy-induced LOX originates. Because we found that PTX treatment increases LOX levels and activity in the lungs in a systemic manner (Fig. 4; Supplementary Fig. S8), we hypothesized that LOX is expressed by immune cells found in the circulation. To investigate this, we assessed the effect of PTX treatment on ECM remodeling in chimeric mice harboring LOX-depleted bone marrow, referred to as bone marrow LOX-depleted (BM-LOX-dep) mice. Chimeric BM-LOX-dep mice were created by transplanting bone marrow from *UBC-Cre^+/−^;LOX^flox/flox^* mice into irradiated wild-type recipient mice, whereas the control mice were transplanted with bone marrow from *UBC-Cre^−/−^;LOX^flox/flox^* mice. Of note, because complete ablation of LOX is not possible due to mouse lethality, donor mice are heterozygous for Cre. Therefore, chimeric mice that were transplanted with bone marrow from *UBC-Cre^+/−^;LOX^flox/flox^* mice are depleted of LOX in bone marrow cells upon tamoxifen administration, but not in the control chimeric mice. Chimeric control and BM-LOX-dep mice were treated with tamoxifen for 5 consecutive days. On day 7, the mice were treated with either PTX or vehicle control and sacrificed 3 days later. Western blot analysis confirmed downregulation of LOX in the bone marrow of BM-LOX-dep mice (Fig. 5A). As expected, control chimeric mice exhibited a significant increase in the level of collagen and elastin in the lungs in response to PTX treatment (Fig. 5B), similar to the effect observed in PTX-treated wild-type mice (Fig. 1C). In contrast, PTX treatment had no effect on collagen levels in the lungs of BM-LOX-dep mice (Fig. 5B), suggesting that BMDCs that express LOX play a major role in pulmonary ECM remodeling following chemotherapy.

To further study this, SCID mice, which lack B and T cells (35), were assessed for pulmonary ECM changes following PTX therapy. SCID mice exhibited no changes in ECM remodeling and no increase in LOX levels in the lungs following PTX treatment (Fig. 5C–G). Consistently, in a PuMA using SCID mice that were pretreated with vehicle control or PTX, no difference in cancer cell seeding in the lungs was observed between the two groups (Fig. 5H and I). These results were also confirmed when using tail-vein injection of EMT6-GFP^+^ cancer cells (2 × 10^5^ cells/mouse) to control or PTX-treated SCID mice that were sacrificed 15 minutes later (Supplementary Fig. S10A and S10B) as well as in an *in vivo* experimental metastasis assay (Supplementary Fig. S10C–S10E). Taken together, these findings strongly suggest that bone marrow cells, and specifically B and T lymphocytes, serve as a major source of chemotherapy-induced LOX that acts in the lungs to promote ECM remodeling.

### Chemotherapy induces LOX expression in CD8^+^ T cells

**Figure 6.**
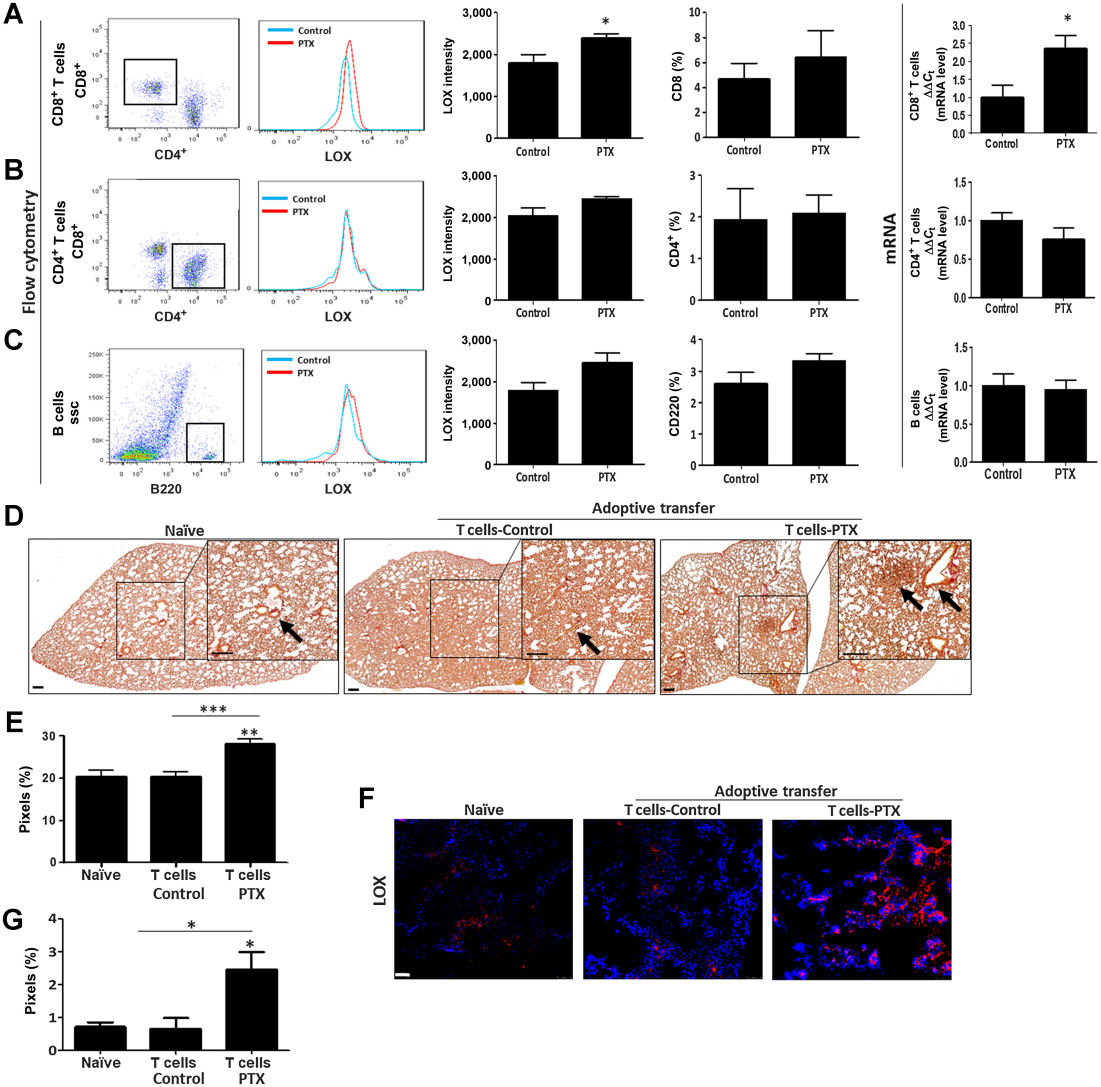
LOX-expressing CD8^+^ T cells promote pulmonary ECM remodeling in response to chemotherapy. Tumor-free, 8- to 10-week-old BALB/c mice (*n* = 5 mice/group) were treated with PTX or vehicle control. After 72 hours, lungs and spleens were removed. **A–C,** LOX intensity presented by histogram and bar graph was assessed by flow cytometry (left), and LOX mRNA levels were assessed by RT-qPCR (right) in CD8^+^ T cells (**A**), CD4^+^ T cells (**B**), and B cells (**C**) in lung tissue. Lymphocyte percentages are shown for the flow cytometry analysis. **D–G,** CD8^+^ T cells, isolated from the spleens of control- and PTX-treated BALB/c mice, were adoptively transferred by injection through the tail vein into naïve SCID mice (2.5 × 10^6^ cells/mouse; *n* = 6–9 mice/group). Control SCID mice were not injected with T cells (naïve). After 72 hours, lungs were removed and analyzed as follows: **D,** Collagen and elastin were detected by Sirius red staining. Representative images are shown in two magnifications. Scale bar, 200 μm. Arrows, positive staining. **E,** Quantification of the percentage of positive pixels per total lung area (*n* > 4 fields/lung) is shown. **F,** LOX was detected by immunostaining. Representative images of LOX staining (red) and nuclear counterstaining (blue) are shown. Scale bar, 50 μm. **G,** A quantification of positive red pixels representing LOX expression per field (*n* > 4 fields/lung) is shown. Statistical significance was assessed by one-way ANOVA followed by Tukey posttest when more than two groups were analyzed or unpaired two-tailed *t* test when only two groups were analyzed. Significant *P* values are shown as *, *P* < 0.05; **, *P* < 0.01; ***, *P* < 0.001 from control or otherwise indicated in the figure.

To identify the specific lymphocyte subset expressing LOX in response to chemotherapy, we harvested spleen, blood, and lungs from mice 72 hours after they were treated with PTX or vehicle control. LOX expression in B- and T-cell subsets was analyzed by flow cytometry. LOX intensity was significantly increased only in CD8^+^ T cells in the lungs and spleens of PTX-treated mice in comparison with control mice (Fig. 6A–C; Supplementary Fig. S11A). In peripheral blood, the percentages of both CD8^+^ and CD4^+^ T cells were increased upon PTX treatment. However, no changes in LOX intensity were detected in these cells (Supplementary Fig. S11B). These results were also confirmed by Western blot, RT-qPCR analyses of LOX expression in CD8^+^ T cells, CD4^+^ T cells, and B cells obtained from spleens of control and PTX-treated mice (Supplementary Fig. S11C). Of note, in the lungs, myeloid lineage cells, fibroblasts, endothelial cells, and platelets did not exhibit significant changes in LOX intensities nor cell percentages (Supplementary Fig. S11D). Of note, increased LOX expression in CD8^+^ T cells but not CD4^+^ T cells and B cells was also observed in lung sections from mice treated with PTX compared with control (Supplementary Fig. S12). These findings and those described in Fig. 4 suggest that CD8^+^ T cells serve as one of the main sources of LOX expressed systemically and in the lungs following PTX treatment.

To further strengthen our results, we performed an adoptive transfer experiment in which CD8^+^ T cells obtained from control- or PTX-treated BALB/c mice were intravenously injected into recipient SCID mice. After 72 hours, the SCID mice were sacrificed and lungs were analyzed for ECM changes and LOX expression. ECM remodeling and LOX expression were enhanced in the lungs of mice adoptively transplanted with CD8^+^ T cells from PTX-treated mice in comparison with mice transplanted with CD8^+^ T cells of control mice (Fig. 6D–G). Of note, adoptive transfer of CD4^+^ T cells or B cells using the same experimental setup did not result in significant changes in pulmonary ECM remodeling as demonstrated by Sirius red staining (Supplementary Fig. S13). Taken together, these results indicate that CD8^+^ T cells promote ECM remodeling in response to PTX therapy, an effect that is associated with an increased expression of LOX systemically and locally in the lungs.

### Blocking LOX activity inhibits metastasis in chemotherapy-treated mice

**Figure 7.**
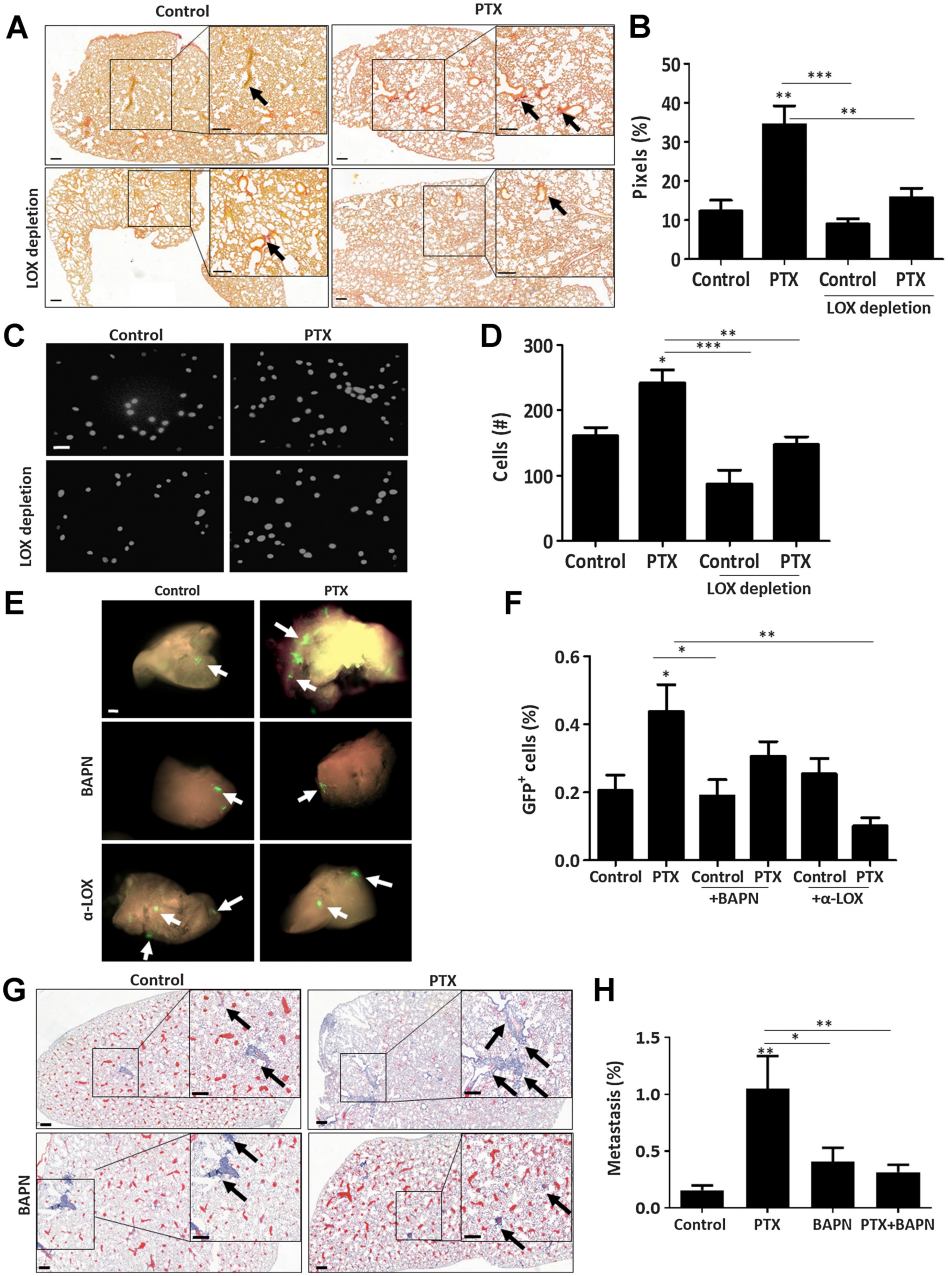
LOX inhibition reduces metastasis in chemotherapy-treated mice. **A** and **B,** Tumor-free, 8- to 10-week-old BALB/c mice were treated with PTX or vehicle control. After 72 hours, blood was obtained and plasma was separated. The plasma was either left unprocessed or depleted of LOX using neutralizing antibodies. Plasma (100 μL/mouse) was injected intraperitoneally into naïve BALB/c mice (*n* = 4–5 mice/group), and after 72 hours, lungs were removed and assessed for collagen and elastin content by Sirius red staining. Representative images in two magnifications are shown in **A**. Arrows, positive staining. Scale bar, 200 μm. Collagen and elastin levels were quantified (**B**) by calculating the percentage of red pixels per total lung area (*n* > 4 fields/lung). **C** and **D,** Collagen-coated plates were incubated with plasma from mice described in **A** and **B** for 4 hours and then washed. EMT6 cells (2 × 10^4^ cells/well) were then seeded onto the plates, and after 15 minutes, nonadherent cells were removed. Adherent cells were stained with DAPI and analyzed by fluorescence microscopy. Representative images are shown in **C**. Scale bar, 50 μm. Quantification of adherent cells is shown in **D**. **E** and **F,** Tumor-free BALB/c mice were treated with vehicle control or PTX in the presence or absence of BAPN or anti-LOX antibodies. After 3 days, EMT6-GFP^+^ cells (25 × 10^4^ cells) were injected through the tail vein for the *ex vivo* PuMA. After 15 minutes, lungs were perfused, excised, and sectioned. Lung sections were cultured for 1 week and analyzed by FluorVivo Mag system. Representative images are shown in **E**. Metastatic foci (green) are indicated by arrows. Scale bar, 200 μm. The percentage of GFP^+^ cells in single-cell suspensions of lung sections was analyzed by flow cytometry (**F**). **G** and **H,** BALB/c mice were implanted with EMT6-F2 cells in the mammary fat pad (*n* = 5 mice/group). When tumors reached 250 mm^3^, mice were treated with vehicle control, PTX, BAPN, or a combination of PTX and BAPN. When tumors in control mice reached 1,000 mm^3^, mice were sacrificed and lungs were analyzed for metastasis by H&E staining. **G,** Representative images are shown in two magnifications. Arrows, metastatic foci. Scale bar, 200 μm. **H,** Lung metastases were quantified per field (*n* > 4 fields/lung). Statistical significance was assessed by one-way ANOVA followed by Tukey posttest when more than two groups were analyzed or unpaired two-tailed *t* test when only two groups were analyzed. Significant *P* values are shown as *, *P* < 0.05; **, *P* < 0.01; ***, *P* < 0.001 from control or otherwise indicated in the figure.

As LOX is upregulated in response to chemotherapy, we next asked whether blocking LOX reverses the effects of chemotherapy-induced ECM remodeling and cancer cell adhesion. To this end, plasma obtained from PTX- or control-treated mice was either left unprocessed or depleted of LOX with neutralizing antibodies. The plasma samples from the four groups were subsequently injected into naïve mice. After 72 hours, the mice were sacrificed, and ECM remodeling in the lungs was analyzed. In contrast to the effect of unprocessed plasma from PTX-treated mice, LOX-depleted plasma from PTX-treated mice failed to promote pulmonary ECM remodeling (Fig. 7A and B), demonstrating the critical role of LOX in this experimental setup. Consistently, in a cancer cell adhesion assay, LOX-depleted plasma from PTX-treated mice failed to enhance EMT6 cancer cell seeding (Fig. 7C and D). To further support these findings, we performed a PuMA in which control- or PTX-treated mice were coadministered with the LOX inhibitor, BAPN, or with anti-LOX neutralizing antibodies. The number of metastatic foci in the lungs was significantly decreased in mice treated with the combination therapies (PTX and BAPN, or PTX and anti-LOX antibodies) in comparison with mice treated with PTX alone (Fig. 7E and F), demonstrating that LOX is necessary for chemotherapy-induced metastasis. Of note, BAPN has no effect on cancer cell viability, as we previously published (23).

We next investigated the role of LOX in chemotherapy-induced metastasis in a clinically relevant breast carcinoma model. To this end, mice were orthotopically implanted with EMT6-F2 cells, a metastatic variant of the EMT6 cancer cell line. When tumors reached a size of 250 mm^3^, mice were treated with vehicle control, PTX, BAPN, or a combination of PTX and BAPN. At endpoint, mice were sacrificed and metastatic foci in lungs were quantified. PTX-treated mice exhibited a significant increase in the number of metastatic foci in the lungs in comparison with control mice, whereas BAPN treatment alone had no effect. Importantly, the number of metastatic foci in the lungs was significantly reduced in mice treated with the combination of PTX and BAPN, in comparison with mice treated with PTX alone (Fig. 7G and H). Our results confirm that chemotherapy induces rapid ECM remodeling in the lungs in an LOX-dependent manner, thereby enhancing cancer cell seeding and metastasis. Furthermore, they demonstrate that LOX inhibition counteracts the metastasis-supporting effects of PTX chemotherapy.

## Discussion

The current arsenal of breast cancer treatments includes surgery, radiation, chemotherapy, and targeted drugs, which substantially improve survival (36). However, some patients will still develop distant metastasis following treatment. Approximately 30% of women initially diagnosed with early-stage breast cancer will develop metastasis within months to years (37), which is the main cause of death in breast cancer patients (38). A recent study demonstrated that neoadjuvant chemotherapy in breast cancer patients increases the potential of metastasis due to elevated number of TMEM structures within the primary tumor. These structures are composed of macrophages, endothelial and cancer cells that allow the escape of cancer cells from the primary tumor site to peripheral blood, thereby contributing to metastasis (20). In addition to these presumably local effects of chemotherapy, preclinical studies demonstrated that systemic host protumorigenic effects are generated in response to chemotherapy, which then contribute to metastasis (17). For example, we demonstrated that macrophages from PTX-treated mice promote lymphangiogenesis in primary breast cancer models and support pulmonary metastasis (39). In addition, circulating extracellular vesicles originated from primary breast cancer cells support metastasis via the formation of premetastatic niche (40). Our current study provides an additional mechanism to explain the contribution of chemotherapy to pulmonary metastasis. We show that chemotherapy induces the expression of LOX in CD8^+^ T cells, which in turn leads to ECM remodeling in the lungs, ultimately facilitating cancer cell seeding and metastasis. We also show that these effects are independent of the tumor, as many of the experiments were performed on tumor-free mice. Thus, we demonstrate a novel host-mediated mechanism by which PTX treatment contributes to metastasis.

The protein composition of the ECM includes several types of collagen, fibronectin, laminin, and elastin (8). ECM remodeling in cancer affects a number of cellular and physical processes including cell signaling, ECM stiffness, and cancer migration, and invasion (41). Tumor ECM remodeling involves changes in ECM protein content, posttranslational modification, proteolytic degradation, and reorganization of fibers (8). Here we focus on posttranslational proteolytic modification. Specifically, we show that collagen is modified within 72 hours after PTX administration. These changes are not associated with its synthesis, but rather with structural modification following chemotherapy. In response to chemotherapy, the ECM becomes more pliable as compared with the untreated control, yet still retains elastic-like responses. The relative reduction in stiffness following chemotherapy may result from microstructural changes, for example in fibers, to effectively “terraform” the premetastatic niche, making it more readily invadable (28). In addition, we show that chemotherapy-induced pulmonary ECM remodeling functionally affects the adhesion of cancer cells to the lungs, in part by increasing focal adhesion signaling. These adhesion activities may involve integrins such as β1, which has been previously demonstrated to support a premetastatic niche (42). Additional studies are required to further elucidate the molecular aspects of ECM remodeling following chemotherapy.

Of particular interest is our finding that ECM remodeling takes place within hours after chemotherapy administration. Previous studies have indicated that fibrosis may promote metastasis and tumor growth (7). The exposure of lungs to radiation or bleomycin increases fibrosis within weeks after therapy, which subsequently promotes metastasis (30). In addition, we have previously demonstrated that surgery in the abdominal region induces rapid changes in pulmonary ECM (23). LOX, a key enzyme contributing to the premetastatic niche (15), promotes the remodeling of ECM within hours after surgery. It does so by increasing collagen cross-linking and the formation of fibrosis (30). Thus, rapid ECM remodeling demonstrated in this study is not restricted to chemotherapy but also found to be relevant in different treatment modalities. It is possible that ECM remodeling occurring at such early stages, before fibrosis takes place, is associated with immune cell colonization, therefore facilitating a premetastatic niche and contributing to cancer cell seeding. Indeed, clustering of immune cells, such as myeloid suppressor cells, requires changes in ECM structure (15, 43), thus chemotherapy may participate in the formation of the premetastatic niches.

We demonstrate that PTX treatment enhances pulmonary ECM remodeling by increasing LOX levels and activity both locally in the lungs, and systemically in the plasma. This led us to study whether BMDCs mediate the systemic effect by expressing LOX in response to chemotherapy. Using chimeric mice harboring LOX-depleted bone marrow cells, we show that BMDCs serve as a major source of chemotherapy-induced LOX, thereby promoting ECM remodeling in the lungs following chemotherapy. These results were also confirmed by experiments using SCID mice that lack T and B lymphoid immune system. In line with these findings, a recent study demonstrated that SCID mice serving as a model for heart failure did not exhibit myocardial fibrosis due to the absence of LOX induction, probably because they lack T cells (44). Thus, similar to the heart, T cells may regulate LOX-induced pulmonary ECM remodeling in our system. We demonstrate that specifically CD8^+^ T cells express higher levels of LOX in the spleen and lungs. We also show that their numbers in peripheral blood and lung tissue are substantially increased upon PTX treatment, indicating that they are one of the main sources of chemotherapy-induced LOX. It is possible that LOX is locally secreted not only by CD8^+^ T cells but also systemically into the circulation, as demonstrated in Figs. 4 and 7. To further strengthen our findings, we show that adoptive transfer of CD8^+^ T cells from PTX-treated BALB/c mice to SCID mice increases pulmonary ECM changes, as opposed to adoptive transfer of CD4^+^ T cells or B cells. In support of these findings, a recent study demonstrated that fibrotic tissue in breast cancer is associated with peritumoral inflammation with the involvement of CD8^+^ T cells. The authors demonstrated that LOX was associated with CD8^+^ T cells in the fibrotic tissue (45), further suggesting a role for LOX-expressing CD8^+^ T cells in ECM remodeling leading to fibrosis.

Our study has several important clinical implications. First, in a clinically relevant breast carcinoma model where tumors spontaneously metastasize to the lungs, we demonstrate that combining PTX with the LOX inhibitor BAPN reverses the prometastatic effect of PTX therapy. These results highlight the potential therapeutic value of LOX inhibition. Second, this study may be relevant to current clinical practice involving immunotherapy based on immune-checkpoint inhibitors, which activate CD8^+^ T cells (46). Specifically, it has been suggested that stiffness of the ECM in tumors may affect immunotherapy outcome by inhibiting the infiltration of immune cells including T cells (47). It is therefore plausible that there is a link between immunotherapy efficacy and the role of CD8^+^ T cells in promoting ECM remodeling. Thus, a thorough investigation of T cells, LOX, and ECM remodeling in the context of immunotherapy is worthy. Third, the mechanism by which PTX induces pulmonary ECM remodeling is not known. Here we suggest that cytotoxic T cells are one of the main sources for LOX in response to chemotherapy. Previous studies demonstrated that bleomycin-induced fibrosis (ECM remodeling) is associated with pulmonary injury and subsequent inflammation (48). Thus, it is possible that activated cytotoxic T cells overexpress LOX, which in turn contributes to ECM remodeling. Fourth, although LOX inhibition via BAPN was found to be clinically toxic (49), additional upstream pathways may be targeted in order to block ECM remodeling. For example, TGFβ is a key regulator of LOX activity and ECM remodeling (14), and therefore its inhibition represents an optional therapeutic approach. Although TGFβ inhibition in cancer did not demonstrate therapeutic effect (50), it is possible that its therapeutic efficacy can be maximized when combined with chemotherapy. Collectively, our study provides translational insights into the growing evidence that chemotherapy promotes metastasis. Accordingly, we propose that counteracting chemotherapy-induced changes in pulmonary ECM may reduce the risk of metastasis specifically in breast cancer patients.

In summary, our study highlights several important points. We show that chemotherapy, such as PTX, may induce pulmonary ECM remodeling and therefore can promote fibrosis, similar to other chemotherapy drugs, e.g., bleomycin (30). We demonstrate that these effects are rapid and take place within 72 hours after chemotherapy administration. Lastly, we show that cytotoxic T cells are one of the main sources of LOX in the periphery and probably locally in the lungs. Our findings warrant further investigation into the effect of other immune altering treatments on ECM remodeling and pulmonary metastasis.

## Authors' Disclosures

J. Haj-Shomaly reports other support from the Ariane de Rothschild Women's Doctoral Program outside the submitted work. T.J. Cooper reports grants from Technion Integrated Cancer Center during the conduct of the study. No disclosures were reported by the other authors.

## Supplementary Material

Supplementary Data supplemental materials and methods and supplemental figures
