# Supplementary material for: T Cells Promote Metastasis by Regulating Extracellular Matrix Remodeling following Chemotherapy
Source: Cancer Res. 2021 Oct 19;82(2):278–91. doi: 10.1158/0008-5472.CAN-21-1012 (PMC7612244; doi:10.1158/0008-5472.CAN-21-1012)
Supplement: Supplementary Data — supplemental materials and methods and supplemental figures [file can-21-1012_supplementary_data_suppsdst1sf1-sf13.pdf]

SUPPLEMENTARY DATA

Haj-Shomaly et. al.,

Supplemental materials and methods

Flow cytometry

Lung and spleen samples were prepared as single cell suspensions as previously described (1,2). Lung, spleen and peripheral blood cells were immunostained with antibodies against CD3-AF700, CD4-BV510, CD8-APCy7, B220-BV605, CD45-FITC, CD11b-PERCP, F4/80-FITC, CD11b-APCy7, Ly6C-PECy7, Ly6G-BV510, CD45-AF700, CD31-APC, CD41-BV421, and αSMA-FITC BioLegend, San Diego, CA or Sigma-Aldrich, St. Louis, MO). The antibody combination was used to identify specific cell types including CD8 T cells (CD8+/CD45+/CD3+); CD4 T cells (CD4+/CD45+/CD3+); B cells (CD45+/B220+); myeloid cell lineage (CD11b+); macrophages (CD11b+/F4/80+); myeloid derived suppressor cells (Ly6C+/Ly6G- or LyC-/Ly6G+); endothelial cells (CD31+/CD45-); fibroblasts (αSMA+/CD45-); and platelets (CD41+). For intracellular staining of LOX, the cells were fixed and permeabilized using cold 70% ethanol. The cells were then immunostained using anti-LOX antibody and Cy3 donkey anti rabbit secondary antibody. Immunostained and GFP-positive cell percentages were assessed by Fortessa flow cytometer and analyzed by Flowjo software. LOX intensity was calculated based on histogram median values.

Immunostaining

Frozen lung tissues were immunostained as previously described (2). Briefly, lungs sections were stained for several ECM components with rabbit anti-collagen type I, rabbit anti-collagen type IV, rabbit anti-fibronectin, and rabbit anti-laminin antibodies. All antibodies were used in a concentration of 1:100 and purchased from Abcam. The expression levels of CD8, CD4 and B220 in lung sections were also assessed using antibodies indicated above (BioLegends), and images were captured by confocal microscopy (Zeiss LSM 700). LOX in lungs was immunostained with rabbit anti-LOX antibody (1:100, Genscript). Cy3-conjugated and DyLight-488-conjugated secondary antibodies were used (1:200, Jackson ImmunoResearch). Nuclei were stained with 6-diamidino-2- phenylindole (DAPI). In some experiments, paraffin-embedded 5µm sections of lungs, liver and spleen were stained with Sirius Red or Masson's Trichrome to detect collagen, several ECM components using the antibodies indicated above, or with hematoxylin and eosin (H&E) to detect metastatic foci. Images

were captured using the Leica DMI6000 B inverted microscope (Leica Microsystems, Wetzlar, Germany). Slides were scanned using Panoramic 250 Flash III scanner (3DHISTECH, Budapest, Hungary).

Organ lysate preparation

Lungs, spleen, liver and tumor tissues were placed in tubes containing RIPA buffer (5M NaCl, 0.5M EDTA pH=8, 1M Tris pH=8, 1% NP-40, 10% sodium deoxycholate, 10% SDS), supplemented with protease inhibitor cocktail (Sigma-Aldrich, St Louis, Missouri, USA). Stainless steel beads (Next Advance, New York, USA) were then added and organ tissues were homogenized using a Bullet Blender tissue homogenizer (Next Advance) according to the manufacturer's protocol. The homogenate was centrifuged at 14,000×g for 15 min at 4°C and supernatant was collected. Protein concentration was determined using Bradford reagent (Bio-Rad Laboratories, CA).

Cell adhesion assay

Cell adhesion to ECM protein-coated substrates was evaluated using a centrifugation assay (3). Briefly, EMT6, 4T1 or MCF7 (20,000 cells/well) were seeded on collagen-coated substrates, that were primed for 4 hours with lung lysates (100μg) or plasma (10%) from control or PTX-treated mice. After a 15 min incubation at 37°C, each well was filled with DMEM media containing 5% FCS, and the plate was covered with sealing tape. The plates were inverted and centrifuged at 1000g for 10 min. The media was carefully aspirated and the remaining adherent cells were stained with DAPI. The adherent cells were counted under a Leica DMI6000 B. All experiments were performed in biological triplicates.

Ex vivo pulmonary metastatic assay (PuMA)

The assay was performed as previously described (4). Briefly, the assay evaluates the potential of tumor cells to seed in the lungs, by injecting tumor cells to mice and 15 min later the lungs are removed, sliced and cultured. Tumor cells bound to lung tissue are then proliferated for a week, demonstrating colonies of single cancer cell seeding. Specifically, EMT6-GFP cells ($2.5x10^{4}$) were intravenously injected to 8-10 week old BALB/c or SCID mice via the tail vein. Fifteen minutes later, mice were anesthetized, and the lungs were perfused. Lungs were filled with heated medium solution (M-199 media, sodium bicarbonate, hydrocortisone, bovine insulin, penicillin/streptomycin and 50% agarose) and then removed. Serial sections (1-2mm in thickness) were gently sliced from each lobe with a

scalpel and incubated on Matrigel covered plates for one week at 37°C. This step allows the ex vivo growth of tumor colonies from single tumor cells that seeded in the lungs in vivo. The lung slices were then analyzed for GFP+ cells using Flourovivo Mag (INDEC Biosystems, Los Altos, CA). In parallel, lung slices were prepared as single cell suspensions and analyzed by flow cytometry.

Second harmonic generation imaging

Frozen blocks of lungs were sliced at a thickness of 100 µm in PBS. The slices were using an upright Leica TCS SP8 MP microscope, equipped with external non-descanned detectors (NDD) HyD and acusto optical tunable filter (Leica microsystems CMS GmbH, Germany). Excitation—SHG signal was excited by a 885 nm laser line of a tunable femtosecond laser 680–1080 Coherent vision II (Coherent GmbH USA). Emission signal was collected using an external NDD HyD detector through a long pass filter of 440 nm. Images were acquired using the galvo stage in a format of 1024 ×1024 (XY) through a HC PL APO 40X/1.10 CS2 objective, and the following parameters: scan speed—400 Hz; zoom—0.75; line average—3; bit depth—16; FOV- X 0.359 µm, Y 0.359 µm; Z step -0.502 µm; pixel size—367.25 nm (XY). Z stacks were acquired using the galvo stage, with 0.502 µm intervals. Data were collected (n=3 mice/group). For quantification measurements, images were analyzed using ImageJ 1.52V. To avoid edge effects (attenuation of the SHG signal at the top and bottom of the section), only the central image of each z stack was included in the quantification. Mean gray value limited to threshold of each image was calculated for each image and averaged over a set of at least five fields of view.

LOX activity assay

LOX activity was evaluated as previously described (5). Briefly, lung lysates were pooled from 5 mice per group (250 µg/mL) and were placed in tubes containing a reaction solution (50 mM sodium borate pH 8.2 and 4 U/mL horseradish peroxidase). Next, substrate mixture solution (50 mM sodium borate pH 8.2, 100 mM, Ampliflu Red (Sigma Aldrich) and 20 mM 1,5-diaminopentane) were added to the mix in order to start the enzymatic reaction. The production of H_2O_2 by LOX results in fluorescent resorufin production, which is detected at excitation of 540 nm and emission of 580 nm wavelengths. The fluorescent reaction was measured every 5 min for 1.5 hr at 37°C using Infinite 200 PRO plate reader.

Rheometry of tissue slices

The assay was performed as previously described (6). Briefly, frozen lung blocks were sectioned into 100 μm thick slices using a cryostat-microtome Leica CM 1950 cryostat (Leica, Germany) to expose the internal structure of the lungs. Rheometry was performed using an AR-G2 magnetic-bearing shear rheometer (TA Instruments, New Castle, Delaware) with a 20-mm diameter, parallel plate fixture. As slices varied in surface area, 50 μl of glycerol was added (Gadot, Israel) to fill the non-containing sample area between the two rheometer plates, ensuring full contact of the sample and preventing sample slippage. Dynamic, oscillatory, shear rheometry experiments were performed with a constant oscillation frequency of 0.5 Hz and with increasing shear strain levels of 1-20% to evaluate the material moduli. The material moduli provide the mechanical response of the samples to the externally applied strains and indicate the samples' (mechano)structure. Specifically, the storage, G', and loss, G'', moduli indicate, respectively, the elastic-like capacity of the sample to store strain energy and the viscous dissipation of the applied energy; a gel-like structure would be more elastic, while a liquid would be viscous. The phase angle, $\tan(\delta)=G''/G'$, indicates if the sample response is more elastic-like or liquid-like, respectively, ranging between 0 and 90 degrees. Results of the rheometry tests were averaged for all tissue slices (5 mice/condition) for control (N=38) and for treated (N=28) samples.

Heparanase activity assay

Preparation of ECM-coated 35mm dishes and determination of heparanase activity were performed as previously described (7). To evaluate heparanase activity in cell extracts, cells ($1x10^6$) were lysed by three freeze/thaw cycles and the resulting cell extracts were incubated with ^{35}S-labeled home-made ECM (prepared as described in (7)) for 18 hours at 37°C. The incubation medium (1 ml) containing sulfate labeled degradation fragments was subjected to gel filtration on a Sepharose CL-6B column. Fractions (0.2 ml) were eluted with PBS and radioactivity was counted in a β-scintillation counter. Degradation fragments of HS side chains are eluted at $0.5< K_{av}<0.8$ (peak II, fractions 15-30) and represent heparanase degradation products. Nearly intact HSPGs are eluted just after the Vo ($K_{av}<0.2$, peak I, fractions 3-15) (7). These high molecular weight products are released by proteases that cleave the HSPG core protein.

Western blotting

A total of 40 µg of lungs' protein was separated by SDS/PAGE gel, and subsequently transferred to nitrocellulose blotting membranes. The membranes were blocked in 5% skim milk in TBST for 1 hr followed by 4°C overnight incubation with the following antibodies: anti-mouse Paxillin (1:1000, Cell Signaling Technology, CST, Denver, MA), anti-mouse Phospho-Paxillin (1:1000, CST), anti-LOX (1:1000, GenScript), anti-MMP9 (1:500, Abcam), anti-collagen type I (1:500, Abcam), anti-collagen type IV (1:500, Abcam), anti-fibronectin (1:500, Abcam), anti-laminin (1:500, Abcam), anti-GAPDH (1:2000, Bioss Antibodies, Wobum, MA) or anti-Actin (1:1000, Sigma-Aldrich) or anti-HSP90 (1:1000, Abcam). GAPDH, Actin or HSP90 were used as loading controls. The bands detected in the blot were assessed by densitometry, and the ratio between the protein of interest and loading control protein was calculated.

References

1. Kan T, Feldman E, Timaner M, Raviv Z, Shen-Orr S, Aronheim A*, et al.* IL-31 induces antitumor immunity in breast carcinoma. J Immunother Cancer **2020**;8
2. Gingis-Velitski S, Loven D, Benayoun L, Munster M, Bril R, Voloshin T*, et al.* Host response to short-term, single-agent chemotherapy induces matrix metalloproteinase-9 expression and accelerates metastasis in mice. Cancer Res **2011**;71:6986-96
3. McClay DR, Wessel GM, Marchase RB. Intercellular recognition: quantitation of initial binding events. Proc Natl Acad Sci U S A **1981**;78:4975-9
4. Mendoza A, Hong SH, Osborne T, Khan MA, Campbell K, Briggs J*, et al.* Modeling metastasis biology and therapy in real time in the mouse lung. J Clin Invest **2010**;120:2979-88
5. Rachman-Tzemah C, Zaffryar-Eilot S, Grossman M, Ribero D, Timaner M, Maki JM*, et al.* Blocking Surgically Induced Lysyl Oxidase Activity Reduces the Risk of Lung Metastases. Cell reports **2017**;19:774-84
6. Barenholz-Cohen T, Merkher Y, Haj J, Shechter D, Kirchmeier D, Shaked Y*, et al.* Lung mechanics modifications facilitating metastasis are mediated in part by breast cancer-derived extracellular vesicles. International journal of cancer Journal international du cancer **2020**;147:2924-33
7. Vlodavsky I, Friedmann Y, Elkin M, Aingorn H, Atzmon R, Ishai-Michaeli R*, et al.* Mammalian heparanase: gene cloning, expression and function in tumor progression and metastasis. Nature medicine **1999**;5:793-802

Supplemental tables and figures

Table S1. List of primers used for RT-qPCR.

Gene	Forward	Reverse
Collagen Type I	GCTCCTCTTAGGGGCCACT	CCACGTCTCACCATTGGGG
Collagen Type IV	CAAAGGCATCAGGGGAATAACT	ATCCGTTGCATCCTGGTAAAC
Fibronectin	ATGTGGACCCCTCCTGATAGT	GCCCAGTGATTTCAGCAAAGG
Laminin	CAGCGCCAATGCTACCTGT	GGATTCGTACTGTTACCGTCACA
LOX	GCCGTATCCAGGTCGGTTC	CAGCCACATAGATCGCATGGT
MMP-9	CTGGACAGCCAGACACTAAAG	CTCGCGGCAAGTCTTCAGAG
Heparanase	ACCGACGACGTGGTAGACTT	GCAGGAGATAAGCCTCTAGCC
TGF-β	CTTCAATACGTCAGACATTCGGG	GCGCTGAATCGAAAGCCCTGTATT
Osteopontin	AGAGCGGTGAGTCTAAGGAGT	TGCCCTTTCCGTTGTTGTCC

SUPPLEMENTAL FIGURES

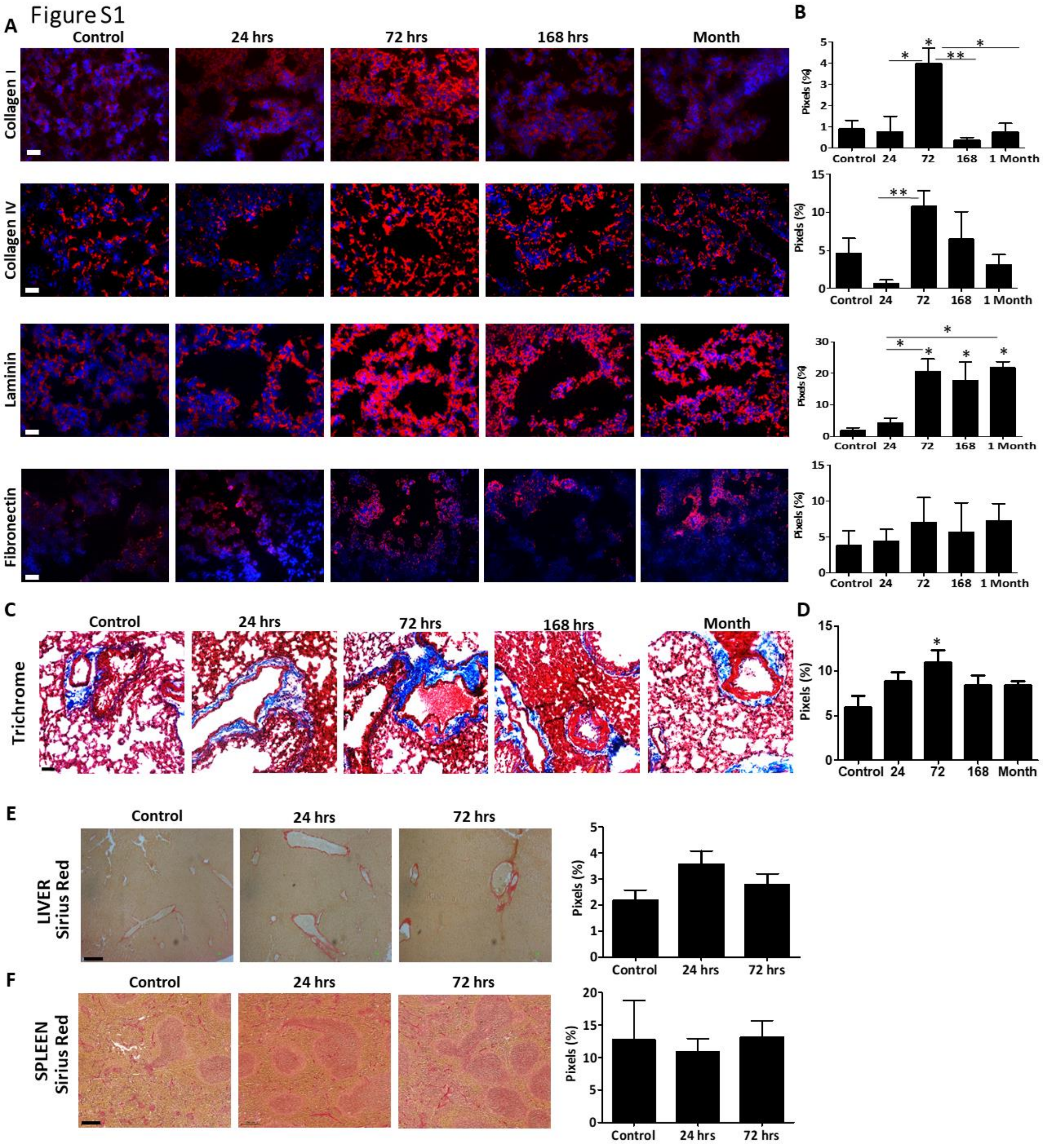

Figure S1: Changes in the expression of ECM components were detected in the lungs but not liver and spleen of mice treated with paclitaxel. Tumor-free, 8-10 week old BALB/c mice were treated with one dose of paclitaxel (PTX) chemotherapy. At the indicated time points, mice were sacrificed and lung (A-D), liver (E) and spleen (F) were removed. Control mice were sacrificed at the 0 hour time point. (A) Lungs were cryosectioned and subsequently immunostained with antibodies against Collagen I, Collagen IV, Laminin and Fibronectin (red). Nuclei were stained with DAPI (blue). Representative images are shown. Bar=50µm. (B) The percentage of red pixels per field was quantified (n>4 fields/lung). In a parallel experiment, paraffin-embedded lung (C-D), liver (D) and spleen (E) sections were stained with Masson's trichrome or Sirius red to assess collagen and/or elastin content. Representative images of organ sections are shown on the left. Bar = 50 µm for the lungs and 200 µm for the liver and spleen. Protein content in sections was quantified by calculating positive pixels per field (n>4 fields/ organ). Statistical significance was assessed by one way ANOVA followed by Tukey post-test were analyzed. Significant p values are shown as * $p<0.05$; ** $p<0.01$ from control or as indicated in the figure.

Figure S2

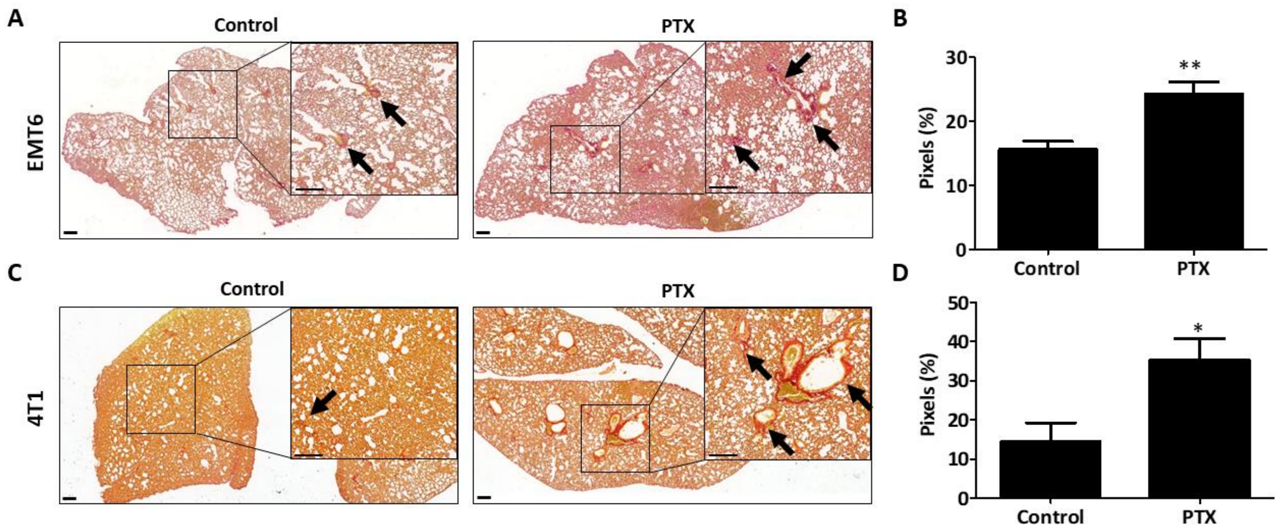

Figure S2: Chemotherapy-induced pulmonary ECM remodeling in breast tumor bearing mice. Eight-to-ten week old BALB/c mice were implanted with EMT6 (A-B) or 4T1 (C-D) murine breast cancer cells ($5x10^5$ cells) into the mammary fat pad (n=4-6 mice /group). When tumors reached $500mm^3$, mice were treated with vehicle control or paclitaxel (PTX). After 72 hours, mice were sacrificed and lungs were removed. (A, C) Lung sections were stained with Sirius red to detect collagen and elastin (red). Representative images are shown in two magnifications. Arrows indicate positive staining. Bar = 200µm. (B, D) Collagen and elastin levels were quantified by calculating the percentage of red pixels per field (n>4 fields/lung). Statistical significance was assessed by unpaired two-tailed t-test when only two groups were analyzed. Significant p values are shown as * $p<0.05$; ** $p<0.01$.

Figure S3

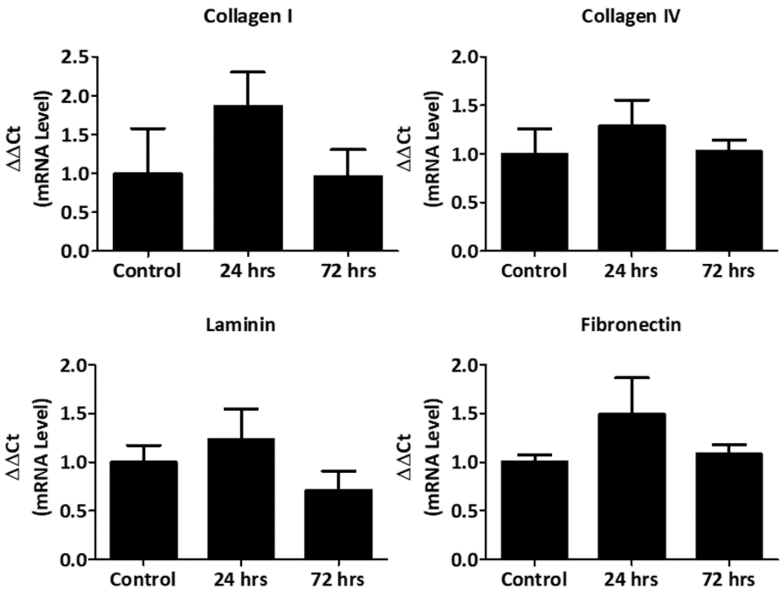

Figure S3: Paclitaxel does not affect mRNA levels of pulmonary ECM components. Tumor-free, 8-10 week old BALB/c mice were treated with paclitaxel (PTX), and lungs were removed at 0 (control), 24, and 72 hours post treatment (n=4-5 mice/group). RNA was extracted from lung tissue. Collagen I, Collagen IV, Laminin, and Fibronectin mRNA levels were assessed by RT-qPCR. No significant changes were found in any of the samples tested as assessed by one way ANOVA followed by Tukey post-test.

Figure S4

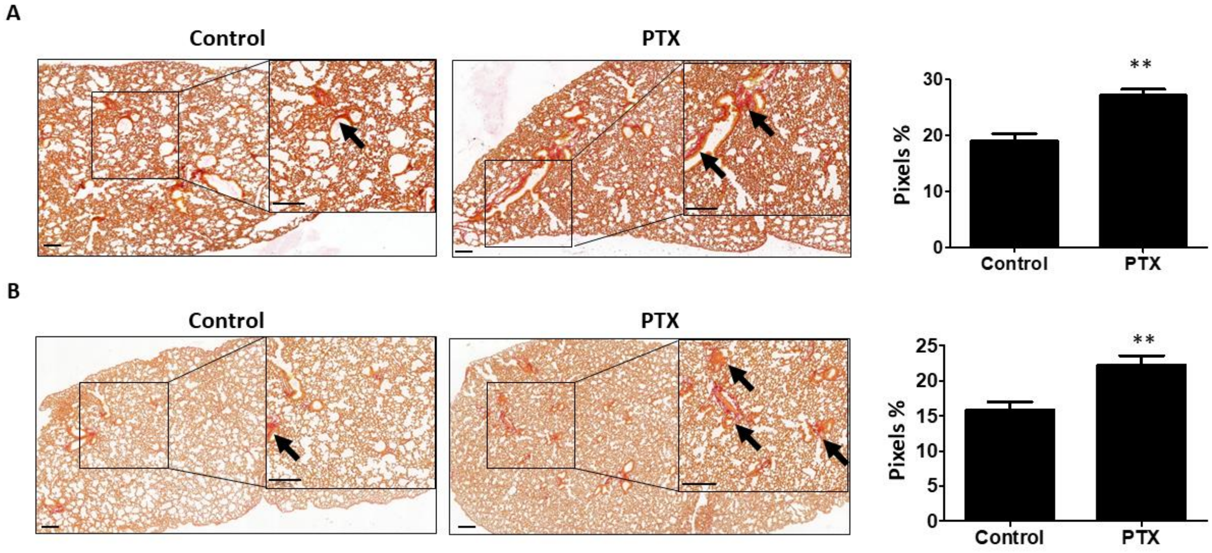

Figure S4: Increased collagen and elastin content in the lungs of mice treated with PTX in different doses or regimes. Tumor-free, 8-10 week old BALB/c mice (n=3-4 mice per group) were treated with (A) paclitaxel (PTX) chemotherapy (25 mg/kg) every 3 weeks for 3 consecutive cycles, or (B) with a lower dose of PTX (10mg/kg) administered once. Control mice were treated with vehicle control. After 72 hours from the last PTX administration, mice were sacrificed and lungs removed. Lung formalin-fixed sections were stained with Sirius red to detect collagen and elastin (red). Representative images in two magnifications are shown. Arrows indicate collagen and elastin staining. Collagen and elastin levels were quantified by calculating the percentage of red pixels per field (n>4 fields/lung). Bar=200µm. Statistical significance was assessed by unpaired two-tailed student t-test. Significant p values are shown as ** p<0.01 from control.

Figure S5

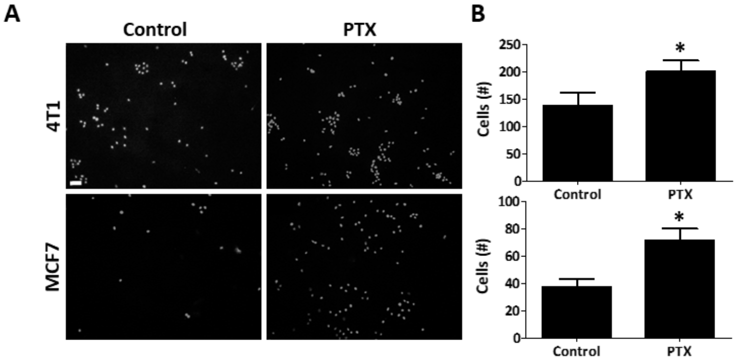

Figure S5: Lung lysates from paclitaxel-treated mice enhance cancer cell adhesion in vitro. Tumor-free, 8-10 week old, BALB/c mice were treated with vehicle control or paclitaxel (PTX). Lungs were removed after 72 hours and lysates were prepared (n= 4 mice/group). Collagen coated plates were pre-treated with lung lysates (100 µg) for 4 hours followed by washing. 4T1 or MCF7 cells ($1x10^5$ cells/plate) were then seeded onto the plates, and after 15 min, non-adherent cells were removed. Adherent cells were stained with DAPI and analyzed by fluorescence microscopy. (A) Representative images are shown; Bar = 75 µm. (B) Quantification of adherent cells is shown (n=3 biological repeats). Statistical significance was assessed by unpaired two-tailed t-test. Significant p values are shown as * $p<0.05$.

Figure S6

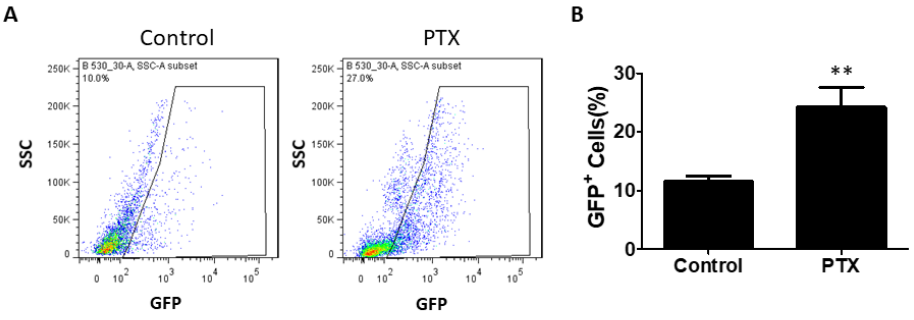

Figure S6: Increased tumor cell seeding in the lungs of mice treated with paclitaxel. Tumor-free, 8-10 week old, BALB/c mice were treated with paclitaxel (PTX) or vehicle (Control) (n=5 mice/group). After 72 hours, EMT6-GFP+ cells ($2x10^5$ cells/mouse) were injected through the tail vein. After 15 min, lungs were perfused, excised and prepared as single cell suspension to evaluate the percentage of GFP+ cells by flow cytometry. (A) Representative dot plots are shown. (B) The quantification of the GFP+ cell percentage was plotted. Statistical significance was assessed by unpaired two-tailed t-test. Significant p values are shown as ** $p<0.01$.

Figure S7

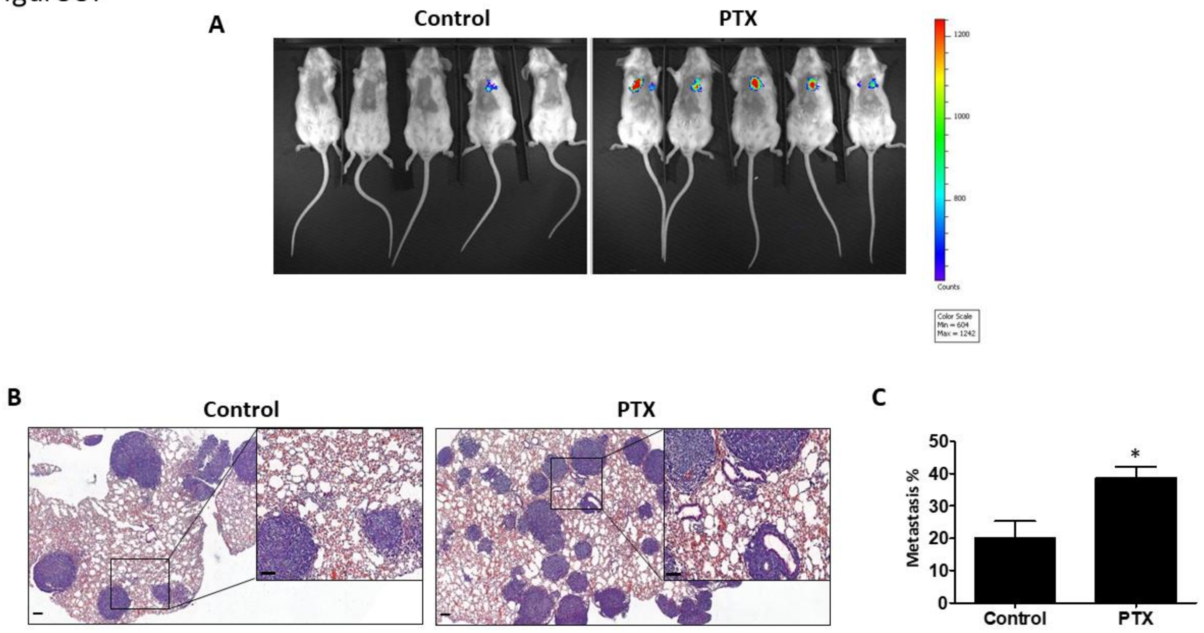

Figure S7: Experimental lung metastasis assay showing PTX-induced metastasis in mice. Eight-to-ten week old, BALB/c mice (n=5 mice /group) were treated with PTX or vehicle control. After 72 hours, the mice were injected with EMT6 breast cancer cells tagged with luciferase ($5x10^4$ cells/mouse) through the tail vein to form lung metastasis. (A) After two weeks, mice were imaged by IVIS to detect pulmonary metastasis by bioluminescence. (B) Subsequently, mice were sacrificed, lungs were removed and stained with H&E to detect metastatic foci. Representative images in two magnifications are shown. Bar = 100µm (C) The quantification of the metastatic foci area from the total lung area was plotted as percentage (n>5 fields). Statistical significance was assessed by unpaired two-tailed t-test. Significant p values are shown as * $p<0.05$.

Figure S8

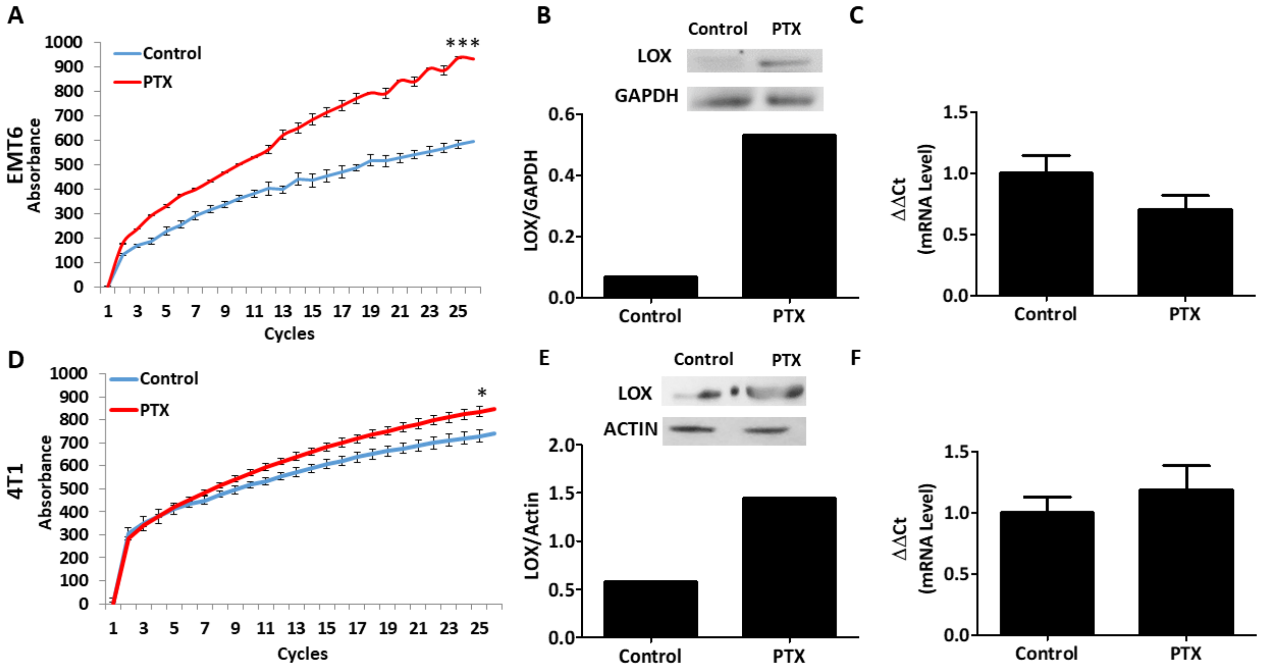

Figure S8: Assessment of LOX protein, mRNA and activity in the lungs of paclitaxel-treated tumor-bearing mice. Eight-to-ten week old, BALB/c mice were implanted with EMT6 ($5x10^5$ cells) or 4T1 ($5x10^5$ cells) cells into the mammary fad pad. When tumors reached $500mm^3$, mice were treated with vehicle control or PTX. After 72 hours, mice were sacrificed and lungs were processed for the evaluation of LOX activity (A, D), protein levels by Western blot (B, E), and mRNA levels by RT-qPCR (C, F). Statistical significance was assessed by unpaired two-tailed t-test. Significant p values are shown as * p<0.05; *** p<0.001.

Figure S9

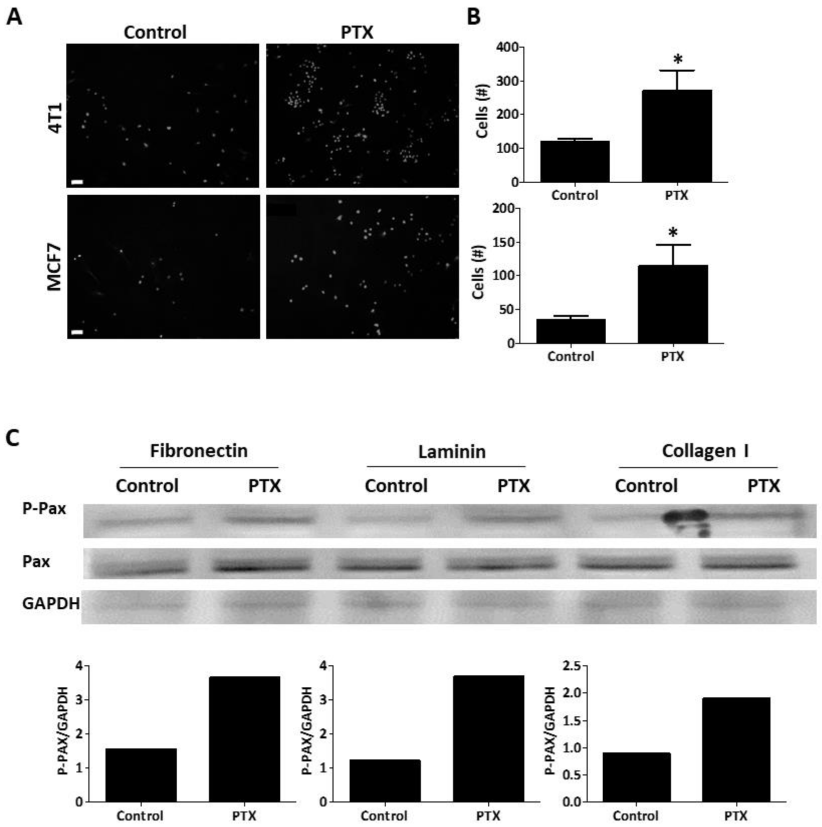

Figure S9: Plasma from paclitaxel-treated mice enhances cancer cell adhesion in vitro. Tumor-free BALB/c mice were treated with paclitaxel (PTX) or vehicle control. After 24 hours, blood was obtained by cardiac puncture and plasma was separated. (A-B) Collagen coated plates were pre-treated with plasma (10%) from control or PTX-treated mice for 4 hours followed by washing. 4T1 or MCF7 cells were then seeded onto the plates, and after 15 min, non-adherent cells were removed. Adherent cells were stained with DAPI and analyzed by fluorescence microscopy. Representative images are shown in (A); Bar = 75 µm. Quantification of adherent cells is shown in (B) (n=3 biological repeats). (C) Plates coated with collagen I, laminin or elastin were primed with plasma from control or PTX-treated mice for 4 hours. MCF7 cells were then seeded onto the plates for overnight. Then, the cells were collected, and lysates prepared. The levels of Paxillin (pax) and phospho-Paxillin (p-pax) in lysates were evaluated by Western blot analysis. GAPDH was used as a loading control. Band densitometry graphs are provided. Statistical significance was assessed by unpaired two-tailed t-test. Significant p values are shown as * $p<0.05$.

Figure S10

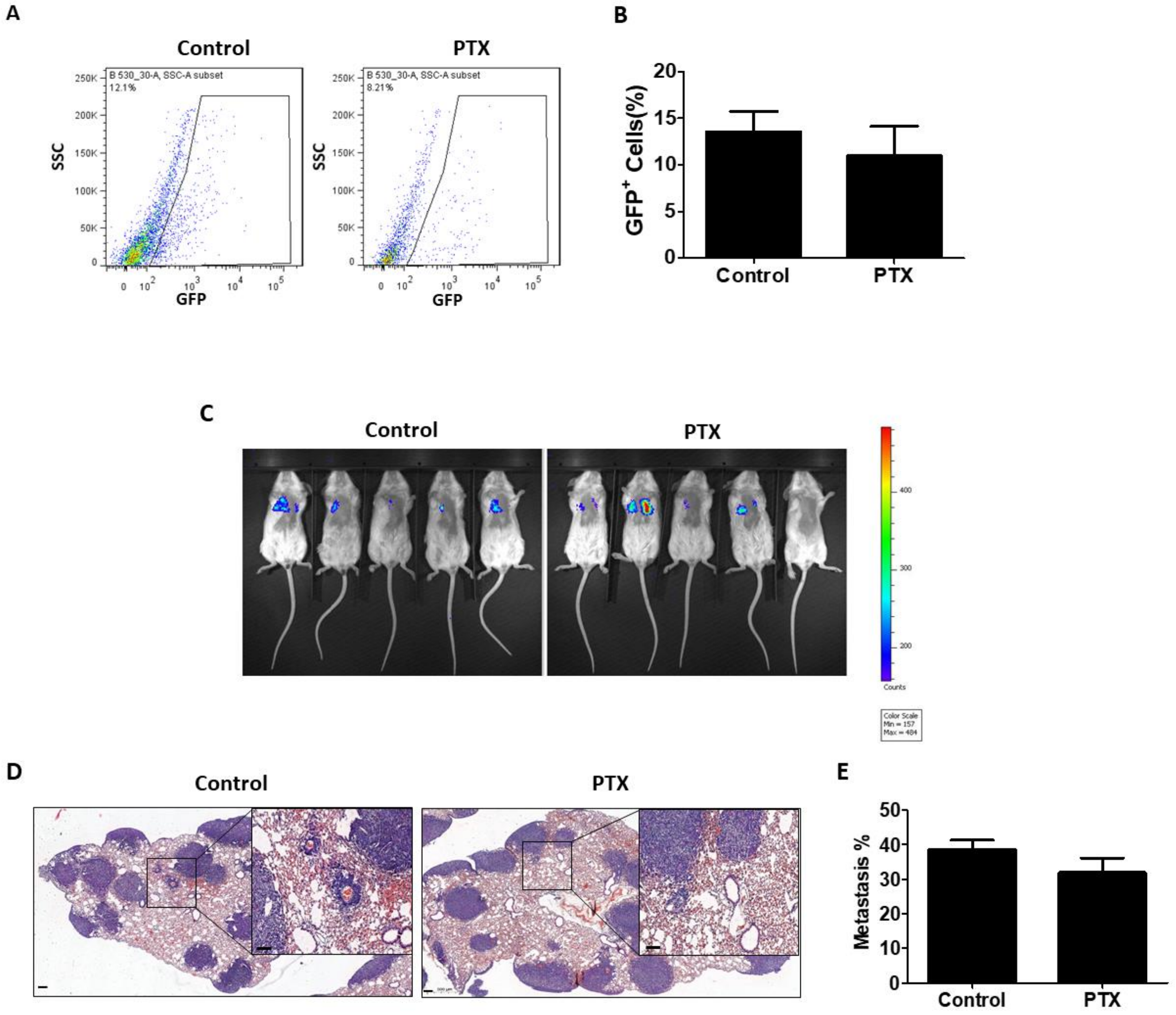
A
Control
PTX
B 530_30-A, SSC-A subset
12.1%
8.21%
SSC
GFP
B
GFP+ Cells(%)
Control
PTX
C
Control
PTX
Counts
Color Scale
D
Control
PTX
E
Metastasis %
Control
PTX

Figure S10: Paclitaxel does not induce tumor cell seeding and metastasis in SCID mice. (A-B)Tumor-free, 8-10 week old, SCID mice were treated with paclitaxel (PTX) or vehicle (Control) (n=5 mice/group). After 72 hours, EMT6-GFP+ cells ($2x10^5$ cells/mouse) were injected through the tail vein. After 15 min, lungs were perfused, excised and prepared as single cell suspension to evaluate the percentage of GFP+ cells by flow cytometry. Representative dot plots are shown (A) along with the quantification of GFP+ cell percentage (B). (C-E) In a parallel experiment, the PTX-treated SCID mice were injected EMT6 cells tagged with luciferase ($5x10^4$ cells/mouse) through the tail vein to form lung metastasis. (C) After two weeks, mice were imaged by IVIS to detect pulmonary metastasis by bioluminescence. (D) Subsequently, mice were sacrificed, lungs were removed and stained with H&E to detect metastatic foci. Representative images in two magnifications are shown. Bar = 100µm. (E) The quantification of the metastatic foci area from the total lung area was plotted and presented as percentage (n>5 fields). Statistical significance was assessed by unpaired two-tailed t-test, and found to be non-significant.

Figure S11

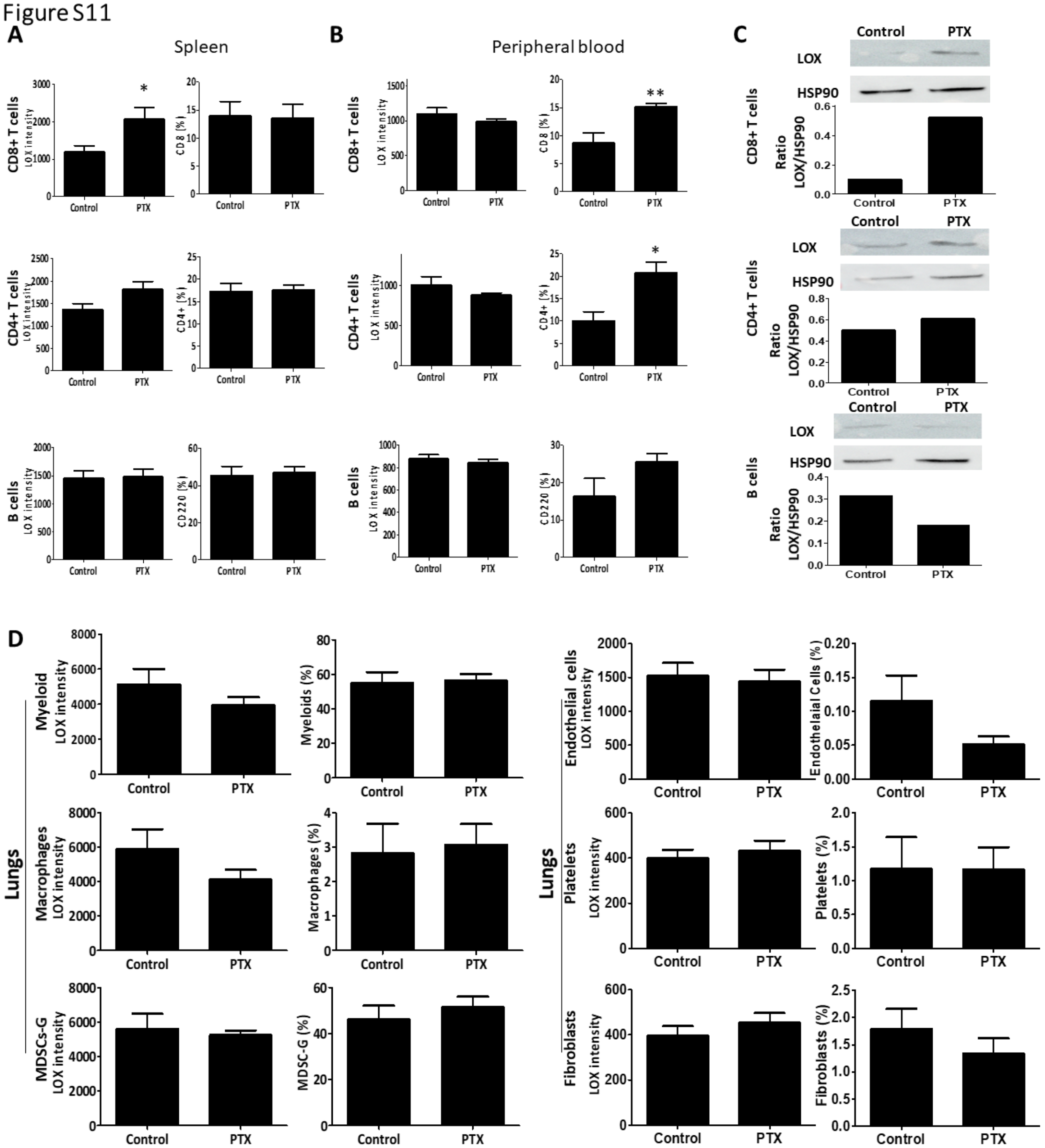
A
Spleen
B
Peripheral blood
C
CD8+ T cells
CD4+ T cells
B cells
LOX intensity
CD8 (%)
CD4+ (%)
CD220 (%)
Control
PTX
LOX
HSP90
Ratio LOX/HSP90
D
Lungs
Myeloid LOX intensity
Myeloids (%)
Macrophages LOX intensity
Macrophages (%)
MDSCs-G LOX intensity
MDSC-G (%)
Endothelial cells LOX intensity
Endothelaial Cells (%)
Platelets LOX intensity
Platelets (%)
Fibroblasts LOX intensity
Fibroblasts (%)

Figure S11: Paclitaxel induces LOX expression in CD8+ T cells. (A-D) Tumor-free, BALB/c mice were treated with paclitaxel (PTX) or vehicle control (n=4-5 mice/group). After 72 hours, peripheral blood (A), spleens (B-C) and lungs (D) were obtained. Flow cytometry was used to assess LOX intensity in the indicated lymphoid cells in peripheral blood (A) and spleens (B). (C) In parallel, CD8+, CD4+ or B220+ (B cells) were isolated from the spleen by sorting, and cell extracts were analyzed for the expression of LOX by Western blot followed by densitometry analysis. (D) Flow cytometry was used to assess LOX intensity in various cell components in the lungs including myeloid cells, macrophages, MDSCs, endothelial cells, platelets and fibroblasts.. Cell percentages are shown. Statistical significance was assessed by unpaired two-tailed t-test. Significant p values are shown as * $p<0.05$; ** $p<0.01$.

Figure S12

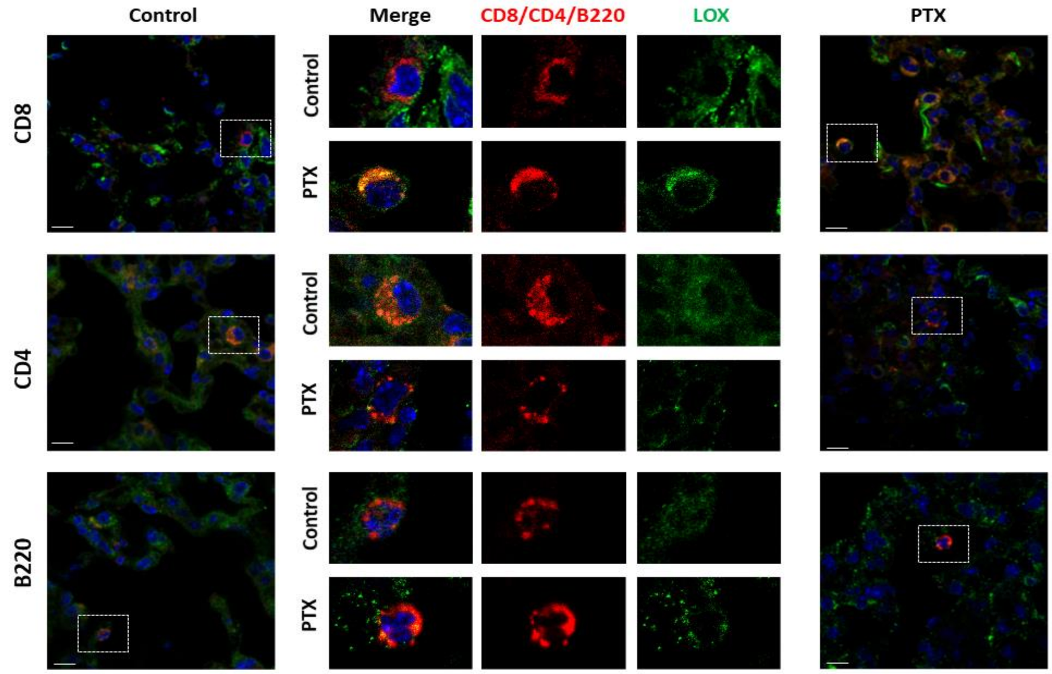

Figure S12: CD8+ T cells in lungs from mice treated with paclitaxel highly express LOX. Eight-to-ten week old BALB/c mice were treated with paclitaxel (PTX) or vehicle (Control). After 72 hours, lungs were removed, cryosectioned, and immunostained with CD8, CD4 or B220 (in red) and LOX (in green). Nuclei were stained with DAPI (blue). Bar = 10 μm. Images were captured by a confocal microscopy system. Merged and separate channels of specific fields as indicated in the images (zoom x4) are also shown.

Figure S13

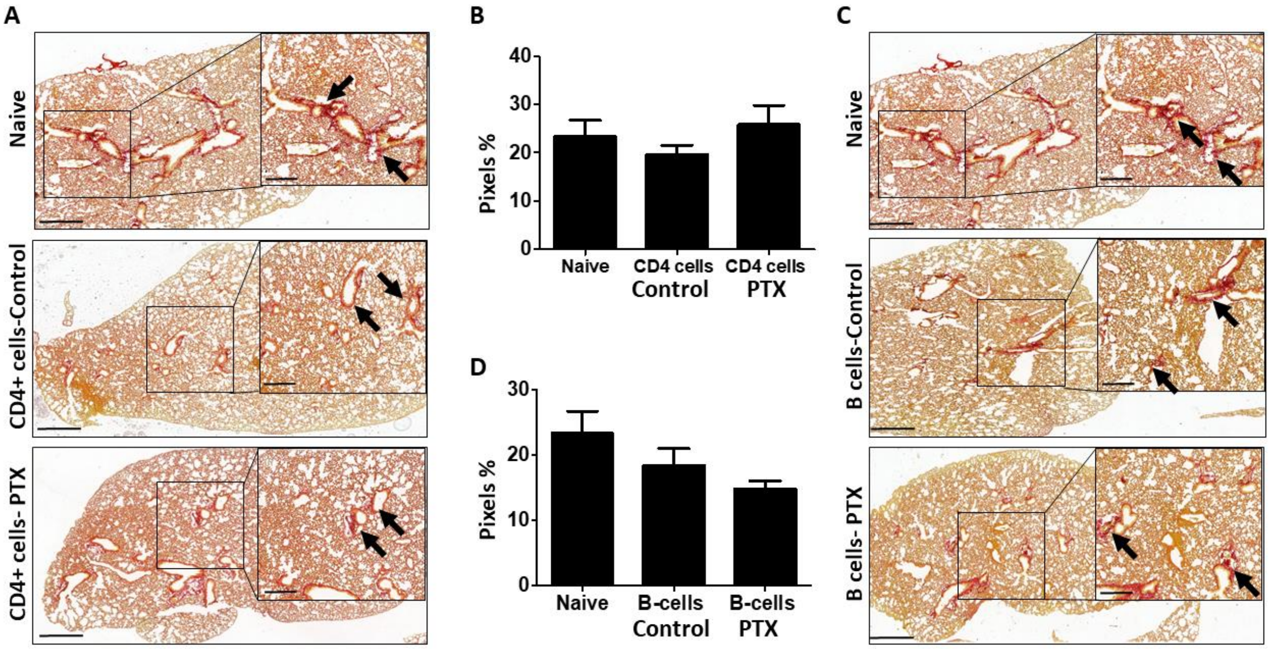

Figure S13: CD4+ T cells and B cells from PTX-treated mice do not affect ECM remodeling. (A-D) CD4+ T cells (A-B) or B cells (C-D) isolated from the spleens of control and PTX-treated BALB/c mice, were adoptively transferred by injection through the tail vein into naïve SCID mice ($2.5x10^6$ cells/mouse; n=6-9 mice/group). Control SCID mice were not injected with cells (naïve). After 72 hours, lungs were removed and analyzed for collagen and elastin content by Sirius red staining. (A, C) Representative images are shown in two magnifications. Bar=200µm. Arrows indicate positive staining. (B, D) A quantification of the percentage of positive pixels per field (n>4 fields/lung) is shown. The results were not statistically significant as assessed by one way ANOVA followed by Tukey post-test.
